# Joint Transcriptomic Analysis of the Effect of Iron Concentration on Piglet Liver and Functional Validation of Iron Regulatory Genes

**DOI:** 10.3390/cimb47100843

**Published:** 2025-10-14

**Authors:** Haiming Qian, Ping Wang, Tengchuan Li, Chunyong Zhang, Jintao Li, Qingliang Wang, Haiyang Ren, Fanyu Jin, Jie Huang, Jun Yao, Hongbin Pan, Rongfu Guo, Qingcong An

**Affiliations:** 1 Yunnan Provincial Key Laboratory of Animal Nutrition and Feed Science, Faculty of Animal Science and Technology, Yunnan Agricultural University, Kunming 650201, China; qhming2001@163.com (H.Q.); mmnwang@163.com (P.W.); zchunyong@126.com (C.Z.); 15924904191@163.com (J.L.); 3113752797@163.com (Q.W.); 1036170166@163.com (H.R.); 13187710828@163.com (F.J.); ynsdyz@163.com (H.P.); 2 College of Animal Science and Technology, China Agricultural University, Beijing 100193, China; ltc583251492@outlook.com; 3 College of Veterinary Medicine, Yunnan Agricultural University, Kunming 650201, China; hjj0909tbb@outlook.com; 4 Yunnan Tropical and Subtropical Animal Virus Disease Laboratory, Yunnan Animal Science and Veterinary Institute, Kunming 650224, China; yaojun_joshua@hotmail.com

**Keywords:** iron, Duroc piglets, liver, transcriptomic analysis, functional genes

## Abstract

Iron plays a key role in oxygen transport, hematopoiesis, and hypoxia adaptation. This study aimed to explore the dynamic response mechanism of the iron regulatory network and key genes in Duroc piglets. Eighteen weaned piglets were randomly divided into three dietary intervention groups: low iron (0 mg/kg), conventional (100 mg/kg), and high iron (200 mg/kg). Transcriptomics technology was used to screen key liver iron regulatory genes under the influence of different dietary iron concentrations, and the expression of related genes was verified using primary pig liver cells. Fasting serum iron metabolism parameters were detected and iron content in organs was quantified. The results show, enrichment analysis highlighted immune–metabolic signaling, including *NF-κB*, *PI3K-Akt*, and *TGF-β*, and a total of 14 candidate genes (such as *FGF21*, *SAA2/3*, *FNDC1*, *ETNPPL*, *TFR1*) were identified. The study observed that these genes showed obvious dosage differentiation and nonlinear patterns. However, findings reflect mRNA-level changes and GO/KEGG over-representation, protein-level validation is planned in follow-up studies. Through the integration of in vitro and in vivo data, this study discovered new liver genes that may be related to pig iron homeostasis function, providing a theoretical basis for analyzing the regulatory mechanism of piglet iron response.

## 1. Introduction

Iron is vital in biological processes and represents a key component of hemoglobin, which is responsible for transporting oxygen and ensuring the normal functioning of the body’s tissues and organs. Iron is also involved in the composition of many enzymes and plays a key role in energy metabolism, DNA synthesis, and other biological processes [1]. Animals maintain iron homeostasis primarily through two pathways: absorption of exogenous iron, which constitutes approximately 90% of the total iron intake (predominantly as non-heme iron from feed sources, with heme iron contributing ~10%), and recycling of endogenous iron derived from senescent erythrocytes [2]. Non heme iron is reduced to the ferrous form in the intestinal cavity by gastric acid or iron duodenal cytochrome b reductase 1 (DCYTB). The reduced ferrous form is then transported through divalent metal ion transporter 1 (*DMT1*) across the top of intestinal epithelial cells. In the liver, hepatocytes (approximately 80% of the liver mass) are important sites for iron storage and metabolic regulation [3] because these cells can synthesize large amounts of ferritin (iron storage) and produce and release the peptide hormone hepcidin (HEPC), which consists of 25 amino acids and regulates iron absorption and release [4]. HEPC binds to ferroportin (FPN) and mediates the ubiquitination, internalization, and degradation of FPN, thereby limiting the export of iron from the cell into the plasma [5]. Iron modulators and transporters regulate the stabilization of iron concentrations in plasma and extracellular fluid as well as systemic iron levels. HEPC is mainly expressed in the liver and regulated at the transcriptional level by serum iron concentrations. When serum iron levels are elevated, HEPC expression is upregulated, which inhibits iron release, thereby preventing iron accumulation in the body [6,7]. Reduced plasma iron levels result in low transferrin saturation [8] and reduced ferromodulin synthesis. In contrast, systemic regulation of iron homeostasis is regulated through the ferromodulin/ferric transfer protein (FPN) axis [9,10,11], which maintains stable body iron levels and plasma iron concentrations. In addition, post-transcriptional control mediated by the iron regulatory proteins/the iron-responsive elements (IRE/IRP) system is an essential iron regulatory factor [12]. The IRE/IRP system regulates the expression of genes encoding iron metabolism proteins such as *TFR1*, *DMT1*, *HIF-2*, and *FPN1*, thereby maintaining systemic and cellular iron homeostasis through transcriptional and post-transcriptional control mechanisms [13]. Any abnormality in the distribution and content of iron in the body can have deleterious effects on physiological processes; therefore, iron levels need to be tightly regulated to avoid overload [14,15]. In addition, strong association signals have been reported between the fibroblast growth factor 6 gene (*FGF6*) and hemochromatosis, and functional validation showed that *FGF6* regulates iron homeostasis and induces transcriptional regulation of ferredoxin, and its downregulation is associated with dysfunctional iron metabolism in systemic sclerosis and cancer cell [16]. The iron dynamic response lncRNA lncRIM has been shown to promote the expression of *DMT1* and *TFR1*, which are important target genes for iron metabolism regulation downstream of Yes-associated protein (*YAP*) and the reprogramming of cellular iron metabolism by interoperating with neurofibromin 2 (*NF2*), the upstream node molecule of the Hippo pathway [17]. In addition, a previous study investigated the evolution of gastric carcinogenesis by perilipin2 and the biological relationship and intrinsic mechanism between perilipin2 and gastric cancer development and found that perilipin2 could affect the iron apoptosis pathway by regulating *ACSL3*, *ALOX15*, *PRDM11*, *IPO7*, *LC3A*, and other related genes and proteins [18].These research advances provide a new perspective for deepening our understanding of the regulation mechanism of iron metabolism and may also lead to breakthroughs in the diagnosis and treatment of related diseases.

Iron is commonly used as a nutritional supplement in young animals, and iron supplements are available in the form of inorganic iron salts, organic iron salts, and amino acid-chelated iron. In livestock production, piglets are the most intensively studied class of experimental animals for iron supplementation [19], which is primarily because iron-deficiency anemia in piglets leads to growth retardation. Moreover, appropriate iron supplementation has a positive effect on the growth and health of piglets. In contrast, excessive amounts of supplementary iron cannot be metabolized efficiently [20] and may lead to the development of diarrhea and other symptoms [21]. The addition of 150 mg Fe/kg to the basal diet of piglets increases the tissue iron status and activity of iron-containing enzymes [22]. However, the addition of 250 mg/kg increases the occurrence of diarrhea and intestinal fecal coliforms in piglets [23]. Similarly, the addition of high iron levels (750 mg/kg) to calf diets results in alterations in the iron signaling cascade of iron-regulated proteins and iron transporter proteins [24]. Dietary iron supplementation of 800 mg/kg can cause renal impairment and hepatic oxidative stress in broilers and thus represents a health hazard [25]. Thus, proper iron supplementation for weaned piglets positively influences pig production by enhancing feed efficiency, boosting animal resistance, and improving overall performance.

To mimic distinct physiological states of iron homeostasis, we adopted a three-tier dietary design (low/control/high). The control diet provided 100 mg Fe/kg (as-fed), which falls within requirement estimates for nursery piglets (7–25 kg) reported by authoritative guidelines and is widely used as an adequate reference level in porcine studies [26,27,28]. The low-Fe diet contained 0 mg/kg supplemental Fe, a setting commonly used to elicit a marginal iron-limited status in the absence of parenteral iron and to sensitize hepatic iron-responsive pathways [29,30]. The high-Fe diet provided 200 mg/kg supplemental Fe, a supraphysiological exposure frequently employed to model iron loading, ferritin induction, and redox-linked signaling, yet below overt toxicity for short-term feeding in piglets [31,32].

In recent years, advancements in high-throughput sequencing technology have led to the swift rise in RNA-seq as a key system for quantitative transcriptome analysis [33]. Moreover, this technology is of great significance in the field of biology for probing gene expression regulation and revealing potential biological functions, thereby promoting progress in the field [34,35,36]. Accordingly, we aimed to define key iron-responsive genes through transcriptomics and preliminary mRNA validation, generating a mechanistic framework and testable targets to support ensuing functional work and inform breeding practices.

## 2. Materials and Methods

### 2.1. Test Animals and Feeding Conditions

A total of 18 healthy Duroc piglets were obtained. All were 35 days old, and they were evenly split between males and females with comparable body weights. Eighteen weaned piglets were randomly divided into three groups: low-iron group (A, 0 mg/kg), control group (B, 100 mg/kg), and high-iron group (C, 200 mg/kg). Each group consisted of six replicates, with one piglet per replicate. The study was conducted at Yunnan Yandong Hunter Agriculture and Forestry Development Co. Ltd. in Kunming, China, where the piglets were individually housed in separate cages. They were fed their respective experimental diets twice a day at 8 a.m. and 5 p.m. and had unrestricted access to drinking water. Standard immunization and sanitation protocols were maintained throughout the farm. The experiment began with a 5-day adjustment period, followed by a 30-day trial phase. Animals were clinically healthy throughout the study based on routine husbandry inspection.

### 2.2. Feed Preparation

The foundational diets were formulated in accordance with the nutritional guidelines established by the National Research Council (NRC) for Swine (2012 [37] and the Nutritional Requirements for Swine (GB/T39235-2020) [38] (Table 1). In this study, the control group received a standard dose of 100 mg/kg of iron, administered in the form of iron glycinate, while the low-iron group was not provided with any additional iron in their diet. Conversely, the high-iron group was supplemented with 200 mg/kg of iron, also in the form of iron glycinate (composed of glycine ≥ 21% and Fe^2+^ ≥ 17%), which was sourced from Hangzhou Huineng Animal Medicine Co. (Hangzhou, China).

### 2.3. Sample Collection

Following a 30-day trial period, we drew 5 mL blood samples (a total of 18) from the anterior vena cava using a standard blood collection needle and vacuum tubes without anticoagulant. These samples were left to sit at room temperature (approximately 20 °C) for an hour before being spun down in a centrifuge (Tiangen Biochemical Technology (Beijing) Co., Ltd., Beijing, China) at 3000 rpm for 10 min. We then collected the serum to analyze various biochemical markers and stored it at −20 °C. The samples were then stored in a freezer at −80 °C for 30 days. Subsequently, the 18 piglets were euthanized with intravenous pentobarbital. Liver tissue was collected, placed in cryopreservation tubes, immediately immersed in liquid nitrogen, and stored in a −80 °C refrigerator (all archived liver aliquots stored at −80 °C were exhausted during qPCR optimization and additional biological replicates; no residual tissue remained for protein assays in this study). From each treatment cohort, we chose three liver specimens at random for RNA-seq and qPCR assays. Visceral tissues, such as liver, spleen, kidney, and intestinal tissues, were collected from each group of piglets to determine the tissue iron content (protein assays were not pre-specified in the study protocol and were therefore not performed in the present work).

### 2.4. Indicators and Methods

#### 2.4.1. Growth Performance

At the beginning and end of the trial (day 32), the test pigs were weighed and recorded in the early morning on an empty stomach. During the formal trial phase, the daily feeding quantity and leftover quantity were recorded in real-time, and the initial and final body weights of the test pigs were recorded. Moreover, the average daily gain (ADG), average daily feed intake (ADFI), and feed-to-weight ratio (F/G) values were calculated. The calculation formulas were as follows:

Average daily gain (ADG) = (final weight − initial weight)/number of days(1)

Average daily feed intake (ADFI) = feed consumption/day(2)

Feed-to-weight ratio (F/G) = feed consumption/weight gain(3)

#### 2.4.2. Serum Biochemical Indicators

In this experiment, the total iron-binding capacity (TIBC), serum iron content, glutathione peroxidase (GSH-Px) and malondialdehyde (MDA) content, hypoxia-inducible factor-1 (HIF-1), HEPC, transferrin receptor (TFR), hemoglobin (HB), and erythropoietin (EPO) content were measured in serum. Among them, the TIBC, serum iron content, GSH-Px, and MDA content were determined by UV spectrophotometer using a kit (Nanjing Jianjian Bioengineering Research Institute, Nanjing, China) and HIF-1, HEPC, TFR, HB and EPO were determined using an ELISA Kit (Shanghai Enzyme-Linked Bio-technology Co., Ltd., Shanghai, China).

#### 2.4.3. Determination of Tissue Iron Content

Speciation of Fe^2+^ vs. Fe^3+^ was not performed in this study. The iron content of the liver, spleen, and duodenum tissues was determined by atomic spectrophotometry by accurately weighing 1.2 g (to the nearest 0.0001) of the tissue samples into a crucible, adding 10 mL of nitric acid, mixing the tissue and nitric acid using a glass rod, and leaving the sample covered at room temperature (20–25 °C) overnight. The following day, the samples were heated and digested on a thermostatic hot plate in a fume hood. The solution was digested on the hot plate until it was colorless, transparent, clarified, and no longer discolored (temperature 340 °C). After cooling, the solution was transferred into a 25 mL volumetric flask, washed with ultrapure water in several batches, and then fixed to 25 mL. After filtration, the iron content was determined on a machine using an AASvario 6 atomic absorption spectrophotometer (Analytik Jena AG, Jena, Germany).

#### 2.4.4. Liver Transcriptome Sequencing

Three liver tissue samples were randomly selected from each group and sent to Beijing Bemac Biotechnology Co. for transcriptome sequencing. Fragments per kilobase of exon model per million mapped fragments (FPKM) was used to normalize the transcript or gene expression levels [39]. Differentially expressed genes (DEGs) were screened based on the count value of genes in each sample using DESeq2 software (https://bioconductor.org/packages/release/bioc/html/DESeq2.html, accessed on 25 September 2025) [40], with fold change ≥ 2 and *p* < 0.05 used as the screening criteria. The screened DEGs were also subjected to Kyoto Encyclopaedia of Genes and Genomes (KEGG) and Gene Ontology (GO) functional enrichment analyses to identify the biological functions or metabolic pathways that were mainly affected by the DEGs. Three comparison groups were set up in this study, namely, A (low-iron group vs. control group), B (high-iron group vs. control group), and C (low-iron group vs. high-iron group), with the group listed after the “vs” in the comparison set as the control group. Unless otherwise noted, GO results are reported at the Cellular Component level with FDR < 0.05.

#### 2.4.5. WGCNA Screening for Iron Metabolism Core Genes

Weighted gene co-expression network analysis (WGCNA) operates on the assumption that the underlying gene network follows a scale-free distribution. It defines both the gene co-expression correlation matrix and a neighbor-joining function to map out this network. The algorithm calculates correlation coefficients between different nodes (genes), ultimately building a hierarchical clustering tree. Let each branch of this tree represent a distinct gene module. Within these modules, genes are highly co-expressed, whereas in separate modules, genes show weaker co-expression patterns. We performed co-expression analyses on all genes identified in serum biochemical profiles and sequencing data from Duroc pigs. By comparing the results of these analyses with the DEGs, we aimed to pinpoint the key genes involved in iron metabolism in Duroc pigs under varying iron levels.

#### 2.4.6. qPCR to Detect the Expression of Relevant Genes

Total RNA was extracted using a Total RNA Extraction Kit (Tiangen Biochemical Technology (Beijing) Co., Ltd., Beijing, China) sourced from Tiangen Biochemical Technology (Beijing) Co. For the PCR reactions, primers (Shanghai Jierui Bioengineering Co., Ltd., Shanghai, China) were custom-designed and synthesized by Shanghai Jierui Bioengineering Co., Ltd. The specific primer sequences are shown in Table 2 (qPCR assays quantified mRNA abundance; no protein-level assays were performed in the present study).

#### 2.4.7. Isolation of Piglet Liver Tissue Cells

Cell validation tests were conducted using piglet liver tissue cells obtained from the Key Laboratory of Animal Viral Diseases of the Yunnan Province, Yunnan Academy of Animal Husbandry and Veterinary Science. Piglet liver cells were isolated by a modified shear digestion method: 0–1-day-old piglets were selected, fasted from water for 12 h before the experiment, and then cleaned the next day by spraying alcohol all over the body. They were then bailed out and bled to death from the jugular vein. After flushing blood from the surface, the pigs were placed onto an ultra-clean workbench, and the abdominal cavity was fully disinfected by tincture of iodine. The abdominal cavity was opened, and the liver was put into a beaker, which was filled with 1× MEM containing 1% double antibiotic (penicillin and streptomycin) for 1 h to remove the bacteria and hematocrit. Subsequently, the liver was removed from the beaker, placed on a high-pressure sterile glass plate, crushed by a scalpel, and transferred to the beaker. Then, 0.05% pancreatic enzyme was added to the beaker, which was placed into an incubator for digestion for 30 min at 37 °C. After removing the beaker, the liquid became turbid. A pasteurized pipette was then used to blow the liquid in the beaker sufficiently to disperse the tissue or cells that had been digested. The buffer was rinsed and gently blown repeatedly, after which a small amount of MEM containing double antibiotic and the above blown cell suspension were added to a 50 mL centrifuge tube. The cell suspension was aspirated and filtered through a 70 μM nylon cell strainer to a volume of 50 mL and transferred to a centrifuge tube for low-speed centrifugation. Centrifugation was carried out at 1000 rpm for 15 min. After centrifugation, the supernatant trypsin was discarded. The precipitated cell suspension (including MEM with 1% double antibody) was transferred into cell flasks and cultured in DMEM serum-containing culture medium (10% fetal bovine serum, 1% double antibody) as the basal culture medium at 37 °C in a 5% CO_2_ incubator.

#### 2.4.8. Cellular Iron Deficiency

Porcine liver tissue cells (fifth and sixth generation) were cultured in 96-well plates for 3 days. Then, iron was removed by adding 0, 25, 50, 75, 100, 125, and 150 μmol/L to the basal medium for 24 h to induce iron deficiency stress. A CCK8 kit (Wuhan Sanying Biological Group Co., Ltd., Wuhan, China) was used to detect cell viability, and a Cell Total Iron colorimetric Assay Kit (Wuhan Eliot Bioscience Co., Ltd., Wuhan, China) was used to detect intracellular iron content to determine the optimal iron removal conditions.

#### 2.4.9. Cellular Iron Supplementation

After the DFO treatment, cells were treated with 0, 30, 60, 90, 120, and 180 μmol/L ferric ammonium citrate (FAC) for 12, 24, and 48 h (two 96-well plates for each time point) to observe the effects of FAC on the proliferation and differentiation of porcine hepatocytes. Cell morphology was observed using inverted phase contrast microscopy, and the intracellular iron content was detected by Cell Total Iron colorimetric Assay Kit.

#### 2.4.10. Cellular RNA Extraction

Cells were grouped into six replicates of 0, 30, 60, 90, 120, and 180 μmol/L per treatment concentrations, with one 25T cell vial used per replicate (addition of 5 mL AR). DFO treatment was performed for 24 h, and then the cells were incubated with the corresponding AR concentrations for 48 h. Total cellular RNA was extracted using a sparkjade (Shandong Sikejie Biotechnology Co., Ltd., Jinan, China) kit to determine iron metabolism gene expression.

### 2.5. Statistical Analysis

The data were collated using Excel 2016 and analyzed by one-way ANOVA using SPSS 21.0 software. Duncan’s method was used for multiple comparisons, and the relative expression of genes was calculated using the 2^−ΔΔCT^ method. Data were tested for normality (Shapiro–Wilk) and homoscedasticity (Levene’s test). Between-group comparisons used one-way ANOVA followed by Tukey’s post hoc test. Results are presented as mean ± [SD/SEM]; different lowercase letters denote *p* < 0.05. All results images were plotted using GraphPad Prism 10.1.2 software.

## 3. Results

### 3.1. Piglet Growth Performance

Three groups were designed: A (low iron, 0 mg/kg), B (control, 100 mg/kg), and C (high iron, 200 mg/kg). Table 3 illustrates that an increasing trend occurred in the high-iron group, although the difference was not statistically significant (*p* > 0.05).

### 3.2. Piglet Serum Parameters

Table 4 presents the serum parameters of the piglets. As the dietary iron levels increased, serum EPO levels presented a corresponding rise. Notably, the low-iron group exhibited significantly lower EPO levels compared to the high-iron group (*p* < 0.05). Additionally, serum HIF-1 levels also increased with higher dietary iron, with the low-iron group showing reduced values relative to both the control (*p* < 0.05) and high-iron groups (*p* < 0.05). Notably, the serum iron levels in the low-iron group were greater than those in the control (*p* < 0.05) and high-iron groups (*p* < 0.05). Conversely, the serum TFR levels were lower in the low-iron group than in the control (*p* < 0.05) and high-iron groups (*p* < 0.05). Serum ferritin and soluble transferrin receptor (sTFR) provided system-level protein readouts, complementing hepatic mRNA responses.

### 3.3. Piglet Visceral Iron Content

Table 5 illustrates the iron levels in the visceral tissues of piglets. The findings indicate that the cardiac iron content rose (*p* < 0.05) with higher iron supplementation.

### 3.4. Liver Transcriptome

#### 3.4.1. Sequencing Results and Quality Control

Table 6 presents the anticipated outcomes and quality control metrics. RNA-seq analysis of the eukaryotic reference transcriptome across nine samples yielded a total of 65.93 Gb of clean data, with each sample contributing approximately 5.79 Gb. Notably, the proportion of Q30 bases was impressive and exceeded 93.20%. This suggests that the sequencing data are both comprehensive and high-quality, making them suitable for further analysis. Following the comparative analysis, the clean reads for each sample were aligned with the specified reference genome, achieving a comparison efficiency between 96.06% and 97.18%. Subsequent analyses, including variable splicing prediction, gene structure optimization, and new gene identification, resulted in the discovery of 5258 novel genes, with 984 having functional annotations.

**Figure 1:**
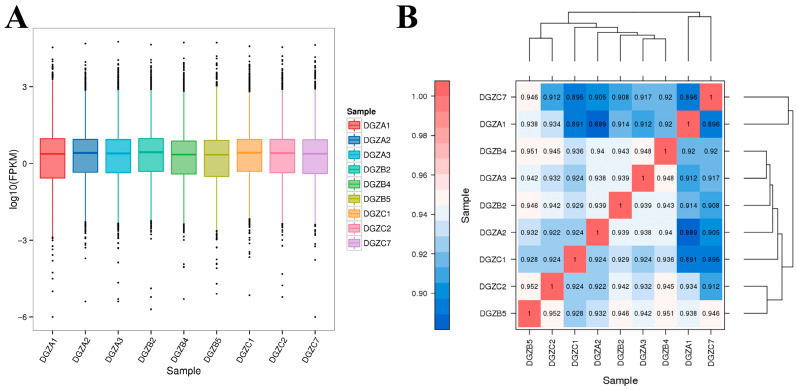
Transcriptomic expression distribution and DEG correlations across iron levels: (**A**) Boxplot analysis of FPKM expression under differential iron concentrations; (**B**) Heatmap of correlation of differentially expressed genes among groups with different iron contents.

The Pearson correlation coefficients between the three biological replicates in each group were greater than 0.89 (Figure 1B), indicating that the samples in the present study were reasonably selected and the experimental data were reliable for further study.

#### 3.4.2. Differential Expression Gene Screening

The low-iron group comprised samples DGZA1–DGZA3; the control group comprised DGZB2, DGZB4, and DGZB5; and the high-iron group comprised DGZC1, DGZC2, and DGZC7. An analysis of DEGs was conducted among three groups with varying levels of iron, and the findings are illustrated in Figure 1A. The horizontal coordinates in Figure 1A represent different samples, and vertical coordinates indicate the logarithmic values of sample expression calculated by the FPKM method. The graph measures the expression level of each sample in terms of the overall dispersion of expression. The larger the value, the higher the expression. All evaluated genes are provided in Supplementary Table S1. The correlation heatmap of differentially expressed genes among different iron content groups is shown in Figure 1B. The value on each color block on the heatmap in Figure 1B represents the correlation between the two samples in the colon block corresponding to the horizontal and vertical axes. The larger the value, the higher the correlation. Each point in Figure 1B represents a gene, and the horizontal and vertical coordinates correspond to the expression of the gene in the samples converted by log2(FPKM+1). The diagonal line and squared value of the correlation coefficient (r) are marked in the figure. The more concentrated the points are near the diagonal, the stronger the gene expression correlation between the two samples.

**Figure 2:**
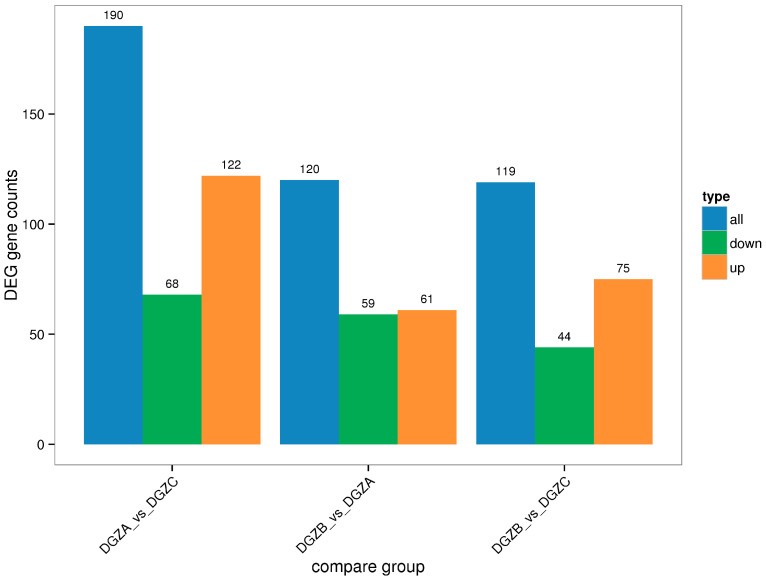
Statistical histogram of differential genes among control groups.

The DEGs in the three treatment groups with different iron levels were assessed relative to that in the control group, and the results are shown in Figure 2. Among them, the low-iron group (DGZA1, DGZA2, DGZA3); the control group (DGZB2, DGZB4, DGZB5); and the high-iron group (DGZC1, DGZC2, DGZC7). Points deviating from the diagonal represent differentially expressed genes (DEGs). The horizontal axis of the differentially expressed gene statistical histogram in Figure 2 represents different DEG groups. Blue represents the total number of DEGs, orange represents upregulated genes, and green represents downregulated genes. The vertical axis represents the number of DEGs. Based on the comparison of sequencing results and the analysis of gene expression calculated by the FPKM method, a total of 190 significant DEGs were obtained between the low- and high-iron groups of Duroc pigs, including 68 downregulated genes and 122 upregulated genes. A total of 120 significant DEGs were obtained between the low-iron group and the control group, including 59 downregulated genes and 61 upregulated genes. A total of 119 differentially expressed genes were obtained between the control and high-iron groups, including 44 downregulated genes and 75 upregulated genes.

#### 3.4.3. Functional Enrichment of DEGs

**Figure 3:**
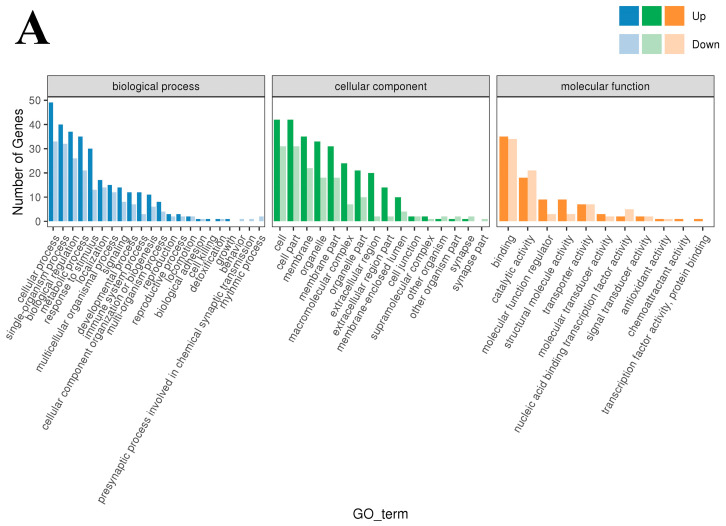

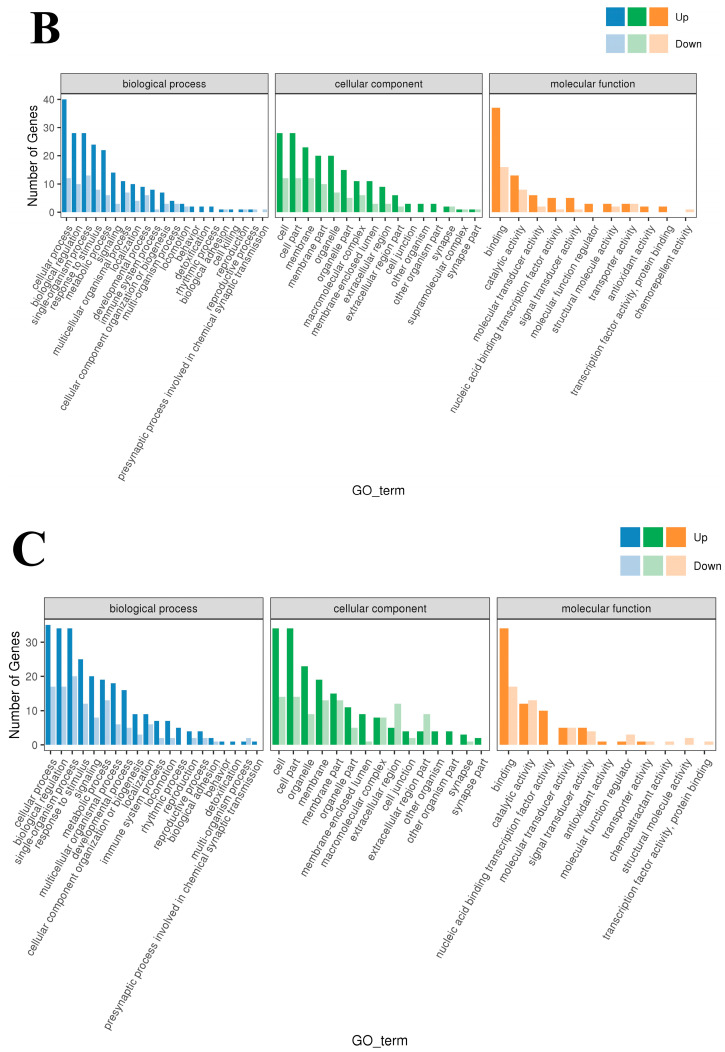
GO enrichment-Cellular Component (CC): (**A**) Classification statistics of GO annotations of DEGs between the low- and high-iron groups; (**B**) Classification statistics of GO annotations of DEGs between the low-iron and control groups; (**C**) Classification statistics of GO annotations of DEGs between the control group and high-iron. Note: Top enriched GO–Cellular Component (CC) terms for DE genes across iron contrasts (FDR < 0.05). Generic labels such as “cell”, “cell part” and “membrane part” are umbrella CC categories grouping specific structures.

To study the biological functions of the DEGs, GO enrichment analysis was performed on the DEGs of the three groups, and the results are shown in Figure 3A–C. Horizontal coordinates represent the GO classification, vertical coordinates represent the number of genes, and different colors represent the different primary classifications to which they belong. Gene Ontology terms shown are Cellular Component categories (CC). Generic labels such as “cell/cell part/membrane part” are umbrella CC terms that group specific subcellular structures. The 190 DEGs obtained from the low- and high-iron groups were categorized into 49 entries. The entry most enriched in DEGs in the “biological process” category was cellular process (GO:0009987), with 49 upregulated and 33 downregulated DEGs; the entry most enriched in DEGs in the “cellular components” category was cell and cell part (GO:0044464; GO:0005623), with 42 upregulated and 31 downregulated DEGs; and the entry most enriched in DEGs in the “molecular function” category was binding (GO:0005488), with 35 upregulated and 34 downregulated DEGs. The 120 DEGs obtained from the low-iron and control groups were enriched in 48 entries. The entry most enriched in DEGs in the “biological process” category was cellular process (GO:0009987), with 40 upregulated and 12 downregulated DEGs; the entry most enriched in DEGs in the “cellular components” category was cell and cell part (GO:0044464; GO:0005623), with 28 upregulated genes and 12 downregulated DEGs; and the entry most enriched in DEGs in the “molecular function” category was binding (GO:0005488), with 37 upregulated and 16 downregulated DEGs. The 120 genes obtained from the high-iron and control groups were enriched in 46 entries. The entry with most enriched in DEGs in the “biological process” category was cellular process (GO:0009987), with 35 upregulated and 17 downregulated DEGs; the entry most enriched in DEGs in the “cellular component” category was cell and cell part (GO:0044464; GO:0005623), with 34 upregulated and 14 downregulated DEGs; and the entry most enriched in DEGs in the “molecular function” category was binding (GO:0005488), with 34 upregulated and 17 downregulated DEGs.

**Figure 4:**
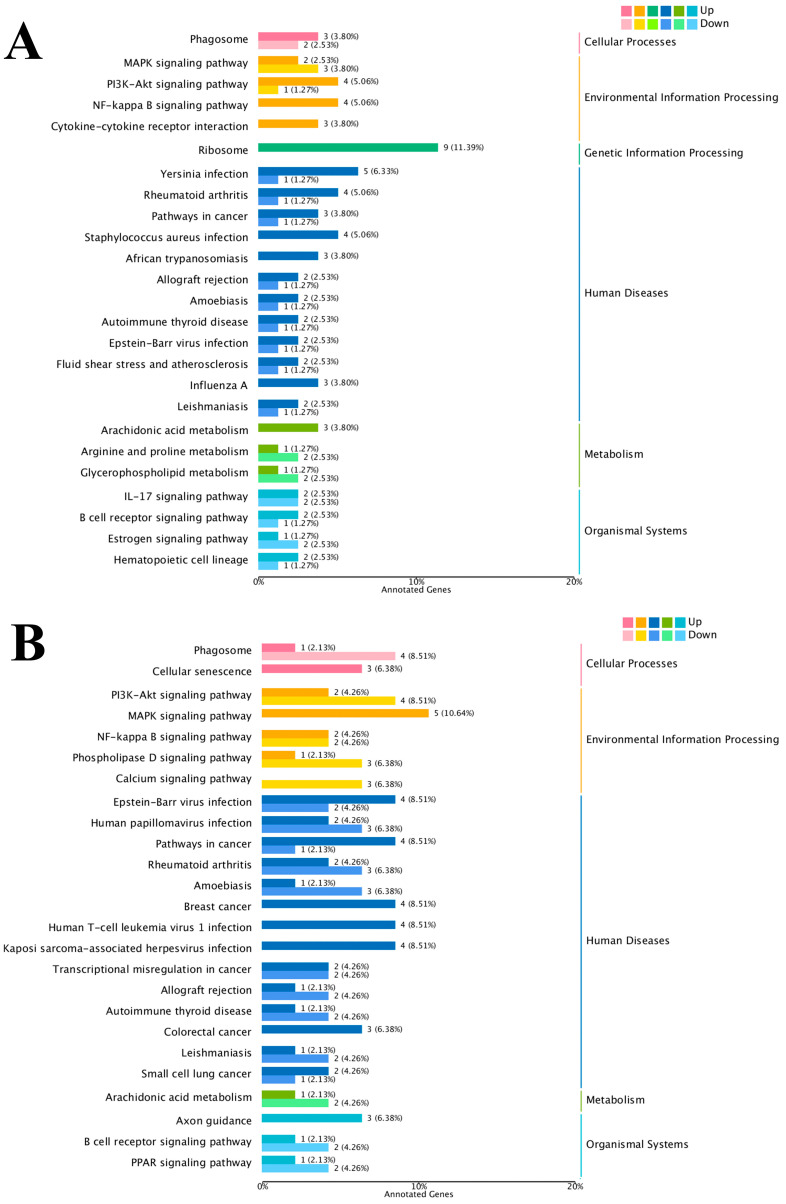

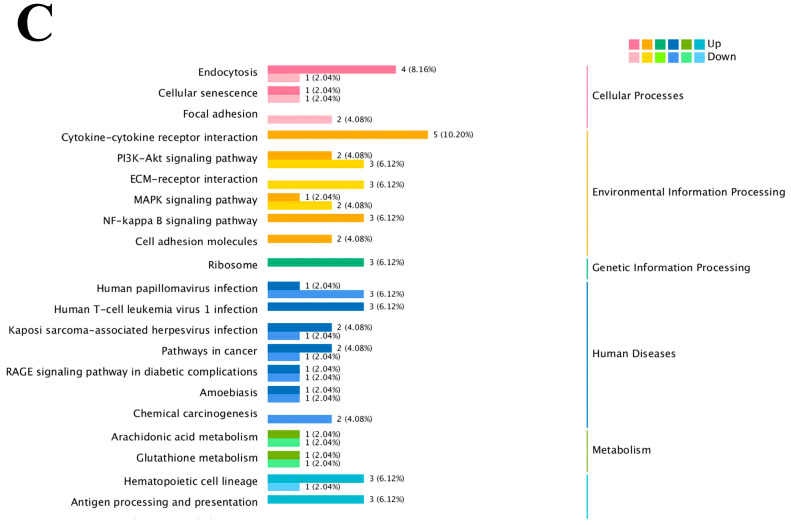
KEGG pathway classification/enrichment of DEGs across iron-status contrasts: (**A**) KEGG classification of DEGs between the low- and high-iron groups; (**B**) KEGG classification of DEGs between the low-iron and control groups; (**C**) KEGG classification of DEGs between the control and high-iron groups. Note: “Viral infection” pathways here reflect host immune signaling overlaps; no clinical signs of infection were observed; *NF-κB* members detected: RELA, TNFAIP3, TRAF6, TLR2, TLR4, NFKB1 [NFKBIA (IκBα) not significant/significant]. Bars/points indicate enrichment at FDR < 0.05.

**Figure 5:**
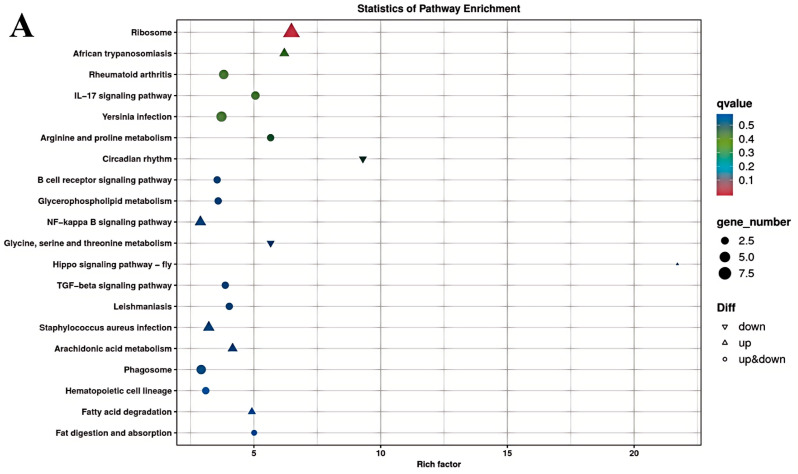

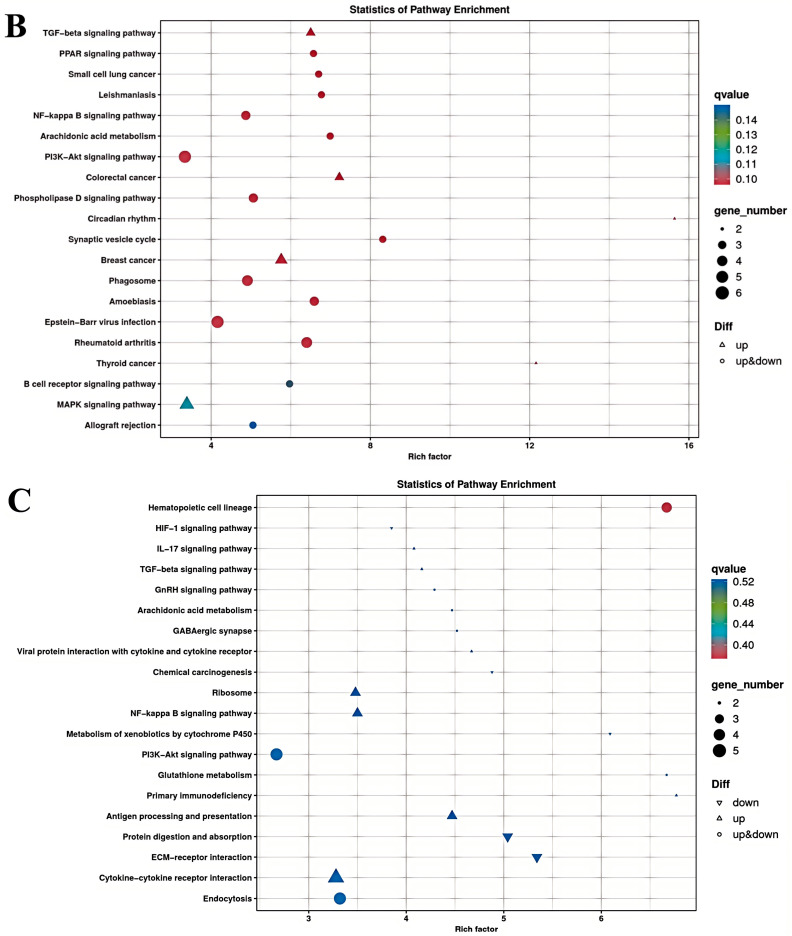
KEGG pathway enrichment of differentially expressed genes between different dietary iron groups (bubble plot): (**A**) KEGG enrichment bubble plots of differentially expressed genes between the low- and high-iron groups; (**B**) KEGG enrichment bubble plots of differentially expressed genes between the low-iron and control groups; (**C**) KEGG enrichment bubble plots of differentially expressed genes between the control and high-iron groups.

The results of the KEGG enrichment analysis for the three groups are shown as KEGG classification entries in Figure 4A–C and bubble diagrams of KEGG pathways in Figure 5A–C. Pathways labeled as “viral infection” in KEGG reflect host immune modules (e.g., *NF-κB*, cytokine signaling) commonly activated in sterile inflammatory contexts. All piglets were clinically healthy during the trial; thus these terms denote host-side signaling overlaps rather than active infections. *NF-κB* pathway. Differential genes mapping to *NF-κB* included RELA, TNFAIP3 (A20), TRAF6, TLR2, TLR4, and NFKB1, with NFKBIA (IκBα) not significant. Unless noted, genes listed were significant at FDR < 0.05. The left vertical coordinate on the KEGG classification map is the name of the KEGG metabolic pathway; the right vertical coordinate represents the name of the first-level classification corresponding to the annotated pathway, and the horizontal coordinate is the number of genes annotated under the pathway, with the number representing a proportion of the total number of annotated genes. Each circle in the KEGG bubble diagram represents a KEGG pathway. The vertical coordinate indicates the name of the pathway, and the horizontal coordinate is the Rich factor, which indicates the ratio of the proportion of DEGs annotated to a certain pathway to the proportion of all genes annotated to that pathway. The larger the Rich factor, the more significant the enrichment level of the DEG in the pathway. The color of the circle represents the q-value, which is the *p*-value after correction for multiple hypothesis testing. The smaller the q-value, the more reliable the significance of the enrichment of DEGs in the pathway. The size of the circle indicates the number of genes enriched in the pathway, with a larger circle indicating more enriched genes. Values closer to the upper right corner of the graph represent pathways with the greatest reference value. The three groups were mainly enriched in the six categories Cellular Processes, Environmental Information Processing, Genetic Information Processing, Human Diseases, Metabolism, and Organismal Systems. Environmental Information Processing was mainly enriched in the MAPK, NF-B, and *PI3K-Akt* signaling pathways, and Metabolism was enriched in the MAPK signaling, lipid metabolism, and amino acid metabolism pathways. DEGs between the low- and high-iron groups were mainly enriched in 15 KEGG pathways (*p* < 0.05), DEGs between the low-iron and control groups were mainly enriched in 33 pathways (*p* < 0.05), and DEGs between the control and high-iron groups were mainly enriched in 10 pathways (*p* < 0.05). The KEGG enrichment results for the three groups, KEGG entry map, and KEGG bubble map were mainly focused on the *PI3K-Akt*, NF-B, and *TGF-β* signaling pathways. Besides *NF-κB/PI3K-Akt/TGF-β*, complement and coagulation cascades, ECM–receptor interaction, and fatty acid metabolism were also enriched (FDR < 0.05). The results indicate that different iron levels induce inflammatory response, immune regulation, cellular transmission, and iron regulation metabolic pathways in piglet livers. These findings are of great significance for related research on iron supplementation.

#### 3.4.4. Weighted Gene Co-Expression Network Analysis

**Figure 6:**
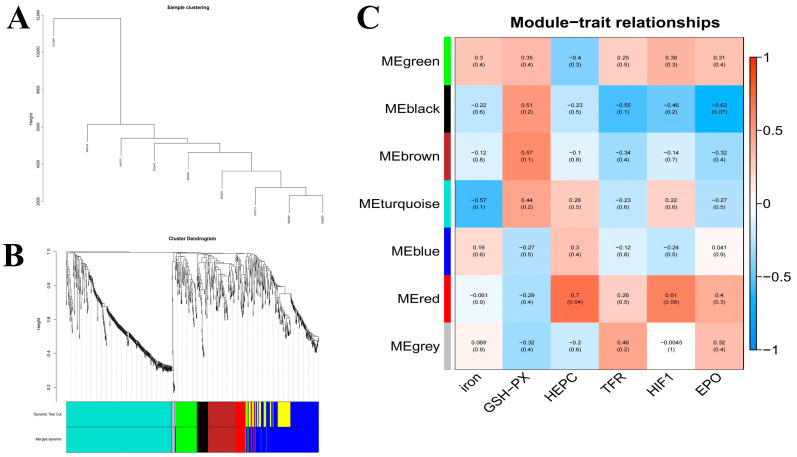
WGCNA of hepatic transcriptomes and module–trait associations across dietary iron levels: (**A**). Weighted gene co-expression network analysis (WGCNA); (**B**). WGCNA co-expression network; (**C**). WGCNA co-expression network and module-trait correlation analysis. Note: Relative mRNA expression of the 14 candidates in liver across dietary iron groups: *FGF21*, *SAA2*, *SAA3*, *ETNPPL*, *RNF125*, *ITIH4*, *TFR1*, and others as indicated.

To more comprehensively assess the interplay between genetic factors and hematopoietic-related serum biochemical markers, we employed weighted gene co-expression network analysis (WGCNA) to build a comprehensive gene co-expression network encompassing all genes and serum biochemical indicators. The analysis was conducted with an expression threshold of 1, a module similarity threshold of 0.25, and a minimum module size of 30 genes. We then performed sample clustering analysis, which leveraged the expression profiles of all genes, with the outcomes illustrated in Figure 6A. The visualization of the co-expression network, depicted in Figure 6B, revealed that genes clustered together and sharing the same color belong to the same module. Ultimately, the analysis yielded six distinct expression modules.

A module correlation heatmap, which was created based on the module color and serum biochemical indexes, is shown in Figure 6C. The closer the correlation between a trait and a module is to the absolute value of 1, the more likely it is that the trait is related to the gene function of the module. In Figure 6C, the horizontal axis represents traits and the vertical axis represents modules. The module values represent correlations, and the numbers in the parentheses of the modules are *p*-values. As indicated by the graphical data, serum iron levels demonstrated the strongest correlation with co-expressed genes in the ME-turquoise module (r = 0.57, *p* = 0.1). Serum HEPC, TRF, and HIF-1 levels were correlated with the MEred module (HEPC, r = 0.7, *p* = 0.04; HIF-1, r = 0.61, *p* = 0.08; TFR, r = 0.26, *p* = 0.5), and serum EPO levels were correlated with the MEblack module (r = 0.63, *p* = 0.07).

**Figure 7:**
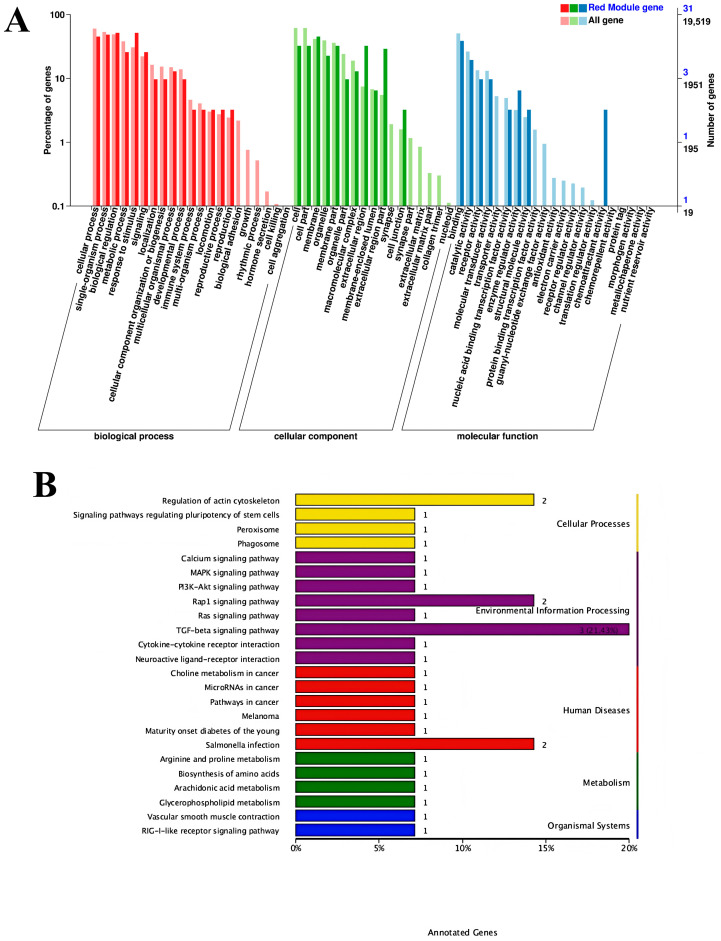
Signaling pathways: (**A**) Gene ontology gene function annotation analysis; (**B**) Kyoto Encyclopedia of Genes and Genomes pathway enriched by the MEred gene module.

The serum iron metabolism parameters ferromodulin, TRF, and HIF-1 presented the highest correlation with the red module, as shown in Figure 6C. Therefore, to better reveal the functions of the co-expressed genes, GO gene function annotation and KEGG pathway enrichment analyses were performed on the MEred gene module. The results of the GO gene function annotation analyses are shown in Figure 7A. The co-expressed genes in the module were enriched in 239 GO terms in the biological process category, with 3 DEGs enriched in biological regulation (biological regulation GO:0065007, biological_process GO:0008150), 4 DEGs enriched in acute-phase response (GO:0006953), and 4 DEGs enriched in cellular component (GO:0006953). The co-expressed genes in the module were enriched in 83 GO terms in the cellular components category, with 6 DEGs enriched in cytoplasmic cytoplasm (GO:0005737), 8 DEGs enriched in integral component of membrane (GO:0016021), and 2 DEGs each enriched in extracellular space receptor activity (GO:0004930), cation binding (GO:0043169), catalytic activity (GO:0003824), binding (GO:0005488), and protein binding (GO:0005515).

The KEGG pathways enriched in the MEred gene module are shown in Figure 7B. A total of 29 KEGG pathways were enriched, including the *TGF-β* signaling pathway (ko04350), regulation of actin cytoskeleton (ko04810), *Salmonella* infection (ko05132), and Rap1 signaling pathway (ko04015). The results in the figure reveal that the regulation of iron metabolism in this experiment is mainly associated with the *TGF-β* signaling pathway.

#### 3.4.5. Screening and Relative Expression of Core Genes

**Figure 8:**
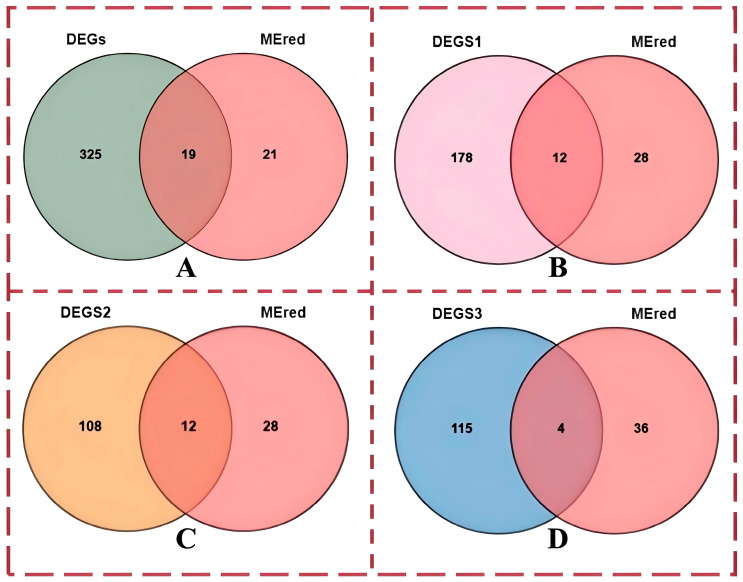
Wayne diagram of genes and differentially expressed genes in the red module of the WGCNA: (**A**) Venn diagram of all differentially expressed genes, genes in the red module, and differentially expressed genes; (**B**) Venn diagram of differentially expressed genes between group A and group C, genes in the red module, and differentially expressed genes; (**C**) Venn diagram of differentially expressed genes between group A and group B, genes in the red module, and differentially expressed genes; (**D**) Venn diagram of differentially expressed genes between group B and group C, genes in the red module, and differentially expressed genes. Note: The “red module” mentioned in the title is the “Mered” module in the figure, which is used to correlate the entire module with the phenotype and select core genes.

We next studied genes related to iron metabolism. The screened DEGs were combined with the WGCNA results to identify common genes between the DEG set and MEred module. A total of 19 genes were obtained, and the results are shown in Figure 8A. The experiment was conducted to screen out the genes that regulate three different iron contents at the same time; therefore, each comparison group was combined with the genes in the red module, and the results of the search are shown in Figure 8B–D. New genes that were not annotated were removed, and differentially expressed core genes and other genes are shown in Table S2. DEGs are all differentially expressed genes in this experiment. DEGS1 is (AvsC); DEGS2 is (BvsA); DEGS3 is (BvsC)

**Figure 9:**
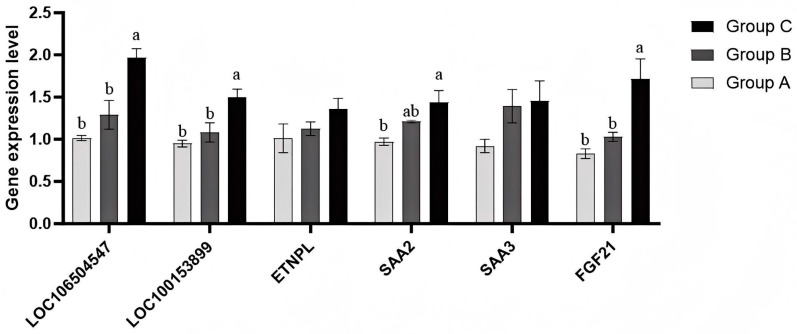
qPCR detection of the expression of each gene. Bars reflect mRNA levels (qPCR); protein abundance was not quantified. NOTE: Group A is the low iron group; Group B is the control group; Group C is the high iron content group. Note: Different lowercase letters indicate significant differences among groups (one-way ANOVA with Tukey’s test, *p* < 0.05); the same letter indicates no significant difference; “ab” denotes no difference from groups labeled “a” or “b”.

To verify whether the differential expression of the screened core candidate genes was consistent with the results of transcriptome sequencing data, some core genes were randomly selected for real-time fluorescence quantitative PCR test. The results are shown in Figure 9 (Group A: low-iron group; Group B: control group; Group C: high iron group.). The results indicated that the expression of each gene is basically consistent with the results of the transcriptome sequencing and WGCNA results. Only the expression of *ENTPL* differed from the WGCNA results, which demonstrates the reliability of the sequencing data and candidate core genes selected in this study.

### 3.5. Iron Metabolism and Gene Expression in Hepatocytes: Chelation, Supplementation, and Transcriptional Control

#### 3.5.1. Hepatocyte Iron Deficiency Treatment

**Figure 10:**
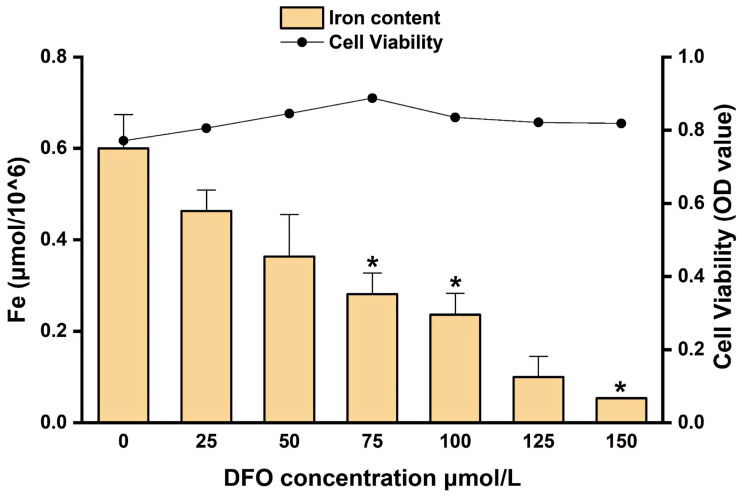
Intracellular iron content and cell viability in hepatocytes treated with different concentrations of DFO. * *p* < 0.05.

The normalized intracellular iron content and cell viability of hepatocytes treated with different concentrations of DFO are presented in Figure 10. As the DFO concentration increased, intracellular iron levels showed a progressive decline. Significant reductions were observed at 75, 100, 125, and 150 μmol/L compared to the 0 μmol/L control (* *p* < 0.05), with the lowest normalized iron content recorded at 150 μmol/L (*** *p* < 0.001). Although 150 μmol/L DFO effectively reduced intracellular iron to the lowest level, a concomitant reduction in cell viability was noted at this concentration. These findings indicate a dose-dependent trade-off between iron chelation efficiency and cell viability. Considering both parameters, 150 μmol/L DFO was selected for subsequent experiments to ensure effective iron depletion. Although this dose may have cytotoxic effects, it produces a robust iron-deficient cell model for downstream iron supplementation assays.

#### 3.5.2. Hepatocyte Iron Supplementation

**Figure 11:**
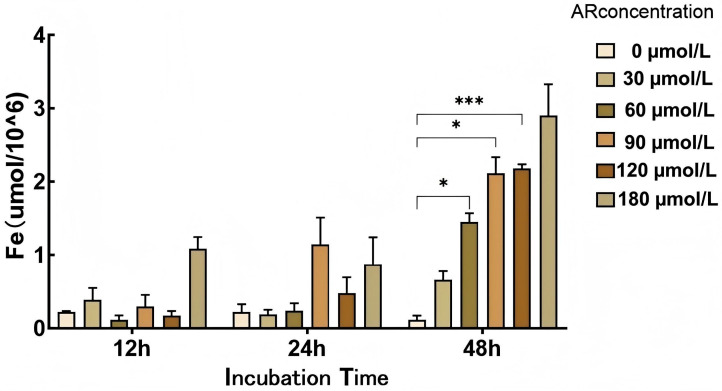
Cellular iron supplementation test. *: *p* < 0.05; ***: *p* < 0.001.

The results presented in Section 3.5.1 revealed that treating hepatocytes with 150 µmol/L DFO for 24 h reduces the intracellular iron content. A cellular iron replenishment test with ammonium ferric citrate (AR) is depicted in Figure 11. The results showed that the intracellular iron content increased gradually. Compared with the values at 12 h and 24 h, the cellular iron content at 48 h was more stable. As the AR content increased, the intracellular iron content also increased, with higher iron contents at 60, 90, and 120 μmol/L than at 0 μmol/L (*p* < 0.05).

#### 3.5.3. Hepatocyte Gene Expression

**Figure 12:**
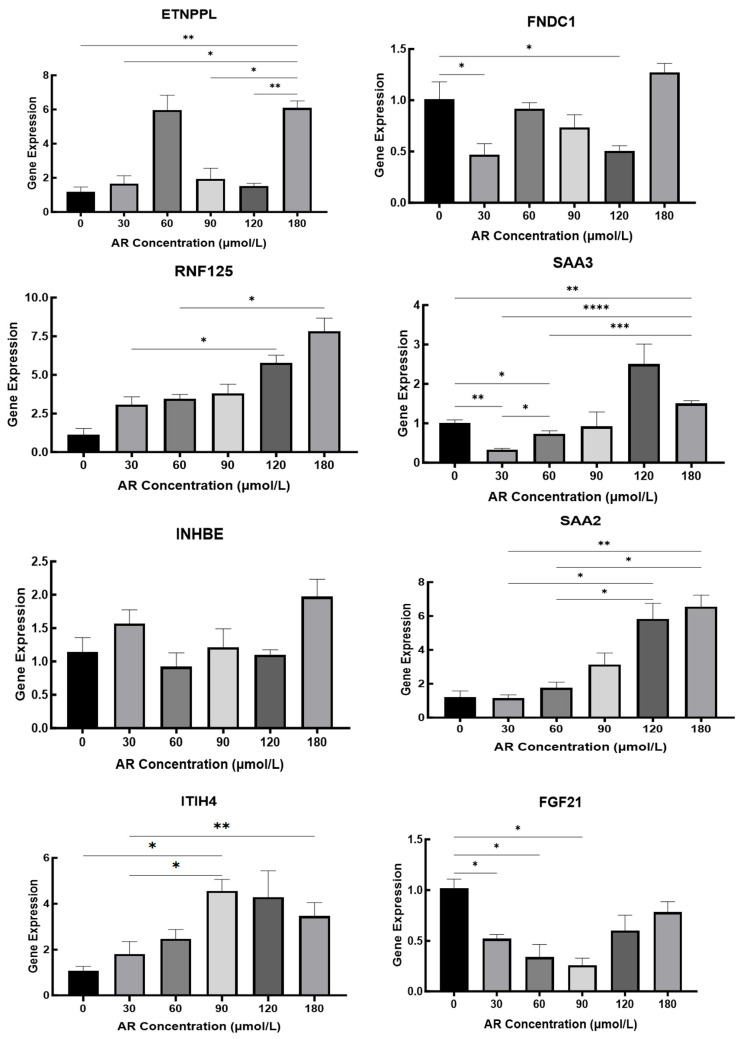
Iron Concentration-Dependent Hepatic Gene Expression Regulation in Duroc Piglets. *, *p* < 0.05; **, *p* < 0.01; ***, *p* < 0.001; ****, *p* < 0.0001.

The results presented in Section 3.5.2 reveal that cellular changes were stable at 48 h; therefore, the total RNA of hepatocytes was extracted after 48 h to detect the expression of related iron metabolism genes. To validate the transcriptome data, we used qPCR to detect the expression of eight key genes at AR concentrations of 0, 30, 60, 90, 120, and 180 µmol/L Figure 12. *RNF125* showed a clear dose-dependent increase, reaching a maximum at 180 µmol/L. The acute phase gene *SAA2* peaked at 180 µmol/L; *SAA3* peaked at 120 µmol/L and then decreased at 180 µmol/L; and *ITIH4* increased sharply in the 60–90 µmol/L range and then decreased slightly. The expression of metabolic regulators was more complex, with *ETNPPL* showing two peaks at 60 and 180 µmol/L and *INHBE* peaking only at the highest concentration. *FNDC1* initially decreased at 30 µmol/L and finally reached its highest level at 180 µmol/L. Notably, *FGF21* expression was highest in the absence of iron supplementation, progressively decreased between 30 and 90 µmol/L, and only partially recovered at 120–180 µmol/L. These different response patterns highlight gene-specific regulatory mechanisms under different iron loads and provide a detailed framework for interpreting the effects of AR on hepatocytes in vitro.

## 4. Discussion

### 4.1. Layered Divergence Between In Vivo and In Vitro Iron Responses

Our integrated design including dietary iron glycinate intervention in piglets in vivo and DFO depletion followed by graded FAC repletion in primary hepatocytes in vitro revealed directional and kinetic divergences in iron-responsive transcription. In vivo, higher dietary iron reduced ADG but not F/G, which is partially consistent with prior reports that moderate oral iron supports growth whereas excess impairs performance [41,42,43,44,45,46,47]; The absence of improvement in F/G corresponds to the differences observed across diet baselines, age, duration, and variance structures in [48,49,50]. At the molecular level, liver RNA-seq and WGCNA revealed enriched inflammatory and metabolic pathways (e.g., *NF-κB*, *PI3K-Akt*, *TGF-β*, and *HIF-1*) and identified 14 candidates. Validation showed that *FGF21* was upregulated in vivo but suppressed at low–mid FAC in vitro; *SAA3* peaked at intermediate FAC and then declined; *SAA2* peaked at higher FAC; and *FNDC1* and *ETNPPL* displayed non-monotonic/dual-peak behaviors. Meanwhile, *TFR1* increased with higher iron in vivo despite its canonical upregulation under deficiency [51,52,53,54,55]. Rather than representing contradictions, these patterns indicate two regulatory layers: a systemic layer in vivo (endocrine, inflammatory, immune, and intercellular cues) and a cell-autonomous layer in vitro (metal-responsive transcription and ROS feedback) that project different response surfaces from the same iron stimulus [56,57,58,59].

### 4.2. Endocrine–Inflammatory Coupling Explains the In Vivo Upregulation of FGF21 and SAA Family

*FGF21* is a hepatokine that regulates nutrient, *PPARα*, and stress inputs, and it can be induced by hepatic iron overload via NRF2/HO-1 [57,58,59]. In piglet livers, higher iron coincided with *FGF21* upregulation, which is consistent with a systemic adaptation to iron-linked oxidative/energetic stress. The acute-phase *SAA* family further illustrates endocrine–inflammatory coupling. In vivo, the *IL-1β/IL-6–STAT3* and *NF-κB* axes robustly drive *SAA* synthesis during inflammation and liver injury [60,61]; while in vitro, the absence of endocrine/cytokine tone constrains *SAA* induction to higher FAC tiers and unmasks gene-specific thresholds. For example, *SAA3* is tightly coupled to *NF-κB* and sensitive to moderate ROS/pro-inflammatory cues (e.g., *TNF-α*), and it peaked at ~120 μM then decreased with the accumulation of IL-10 and self-limiting *NF-κB* inhibitors. However, *SAA2* is more *STAT3/IL-6* dependent (“stronger inflammatory drive”) and peaked at ~180 μM [62,63]. These promoter-architecture differences parsimoniously explain the distinct peak locations we observed.

### 4.3. Metal-Responsive Transcription and ROS Feedback Underpin Non-Monotonic Patterns of FNDC1 and ETNPPL

Cell-autonomous metal sensing and oxidative feedback account for the nonlinear dose–response of several candidates. *FNDC1* followed a “dip–rise–dip–late-rise” trend across FAC gradients, which is consistent with *MTF-1* activation thresholds and ROS-linked reactivation at high iron levels. At 30 μM, labile iron is likely below the *MTF-1* trigger; at 60 μM, crossing the threshold yields a sharp induction; at 90–120 μM, ferritin/FPN buffering plus *NF-κB* inhibitory loops restrain transcription; and at 180 μM, Fenton-driven ROS reengages *MTF-1* and stress-protective programs, restoring a late peak [64,65]. Functionally, *FNDC1* also interfaces with *PI3K/AKT* in regeneration/fibrosis, positioning it at a nexus of stress repair and metabolic remodeling [66]. *ETNPPL* exhibited dual peaks (≈60 and 180 μM), a “hormone-like” biphasic profile wherein moderate ROS transiently augments antioxidant/metabolic reprogramming, feedback suppression ensues, and extreme stress re-activates transcription, thus representing an established motif in stress-adaptation circuits [67,68]. Together, these data support a threshold-and-feedback model for iron-evoked transcription in hepatocytes.

### 4.4. Iron Trafficking and Cell-Type Heterogeneity Reconcile “Stable Total Iron” with Altered Signaling

Despite the lack of significant group differences in total hepatic iron, we observed substantial transcriptional remodeling. This is mechanistically plausible because the liver consist of multiple iron-handling cell types (hepatocytes, Kupffer cells, stellate cells) and subcellular iron pools (ferritin storage, mitochondrial/lysosomal sequestration) that enable micro-redistribution without altering bulk content. Meanwhile, core channels—*TFR1*-mediated uptake, FPN-mediated export, and ferritin storage—can be retuned by inflammatory and oxidative cues to preserve homeostasis at the organ level [69,70,71,72,73,74]. In contrast, the heart displayed a significant rise in iron, which is consistent with more limited buffering capacity and greater sensitivity to plasma iron fluctuations [47,74]. Thus, an organism may prioritize tight regulation in the liver while allowing modest intake-linked variation in peripheral tissues, yielding the observed combination of stable totals and altered. A limitation is that we quantified total iron only; redox speciation (Fe^2+^/Fe^3+^) was not measured and could refine mechanistic links to ROS in future work.

### 4.5. Context Matters: Reconciling Apparent Inconsistencies with Prior Literature (e.g., TFR1, EPO)

Apparent deviations from textbook patterns reflect context-dependent regulation rather than error. First, although *TFR1* is classically *IRP/IRE*-regulated (↑ under deficiency), our in vivo high-iron groups exhibited higher *TFR1*. Prior work shows that iron-induced oxidative stress/inflammation can secondarily upregulate *TFR1* transcription (e.g., in hepatic stellate cells), decoupling it from pure iron-sensing and reframing it as a stress-adaptive readout in certain contexts [51,52,54,55]. Second, while iron availability can suppress EPO under some conditions, clinical observations of iron overload revealed elevated EPO with impaired downstream *STAT5*, which is consistent with EPO resistance and compensatory overproduction rather than effective erythropoiesis [75,76,77] Third, in terms of growth performance, our data showed reduced ADG at higher iron but no F/G decrease, which we interpret as partial agreement with certain previous studies [41,42,44,46,47] and methodological/context differences with others [48,49,50] (diet background, age, duration, *n*, variance) rather than contradictions. According to this framing, our dataset extends the prior literature by mapping when systemic endocrine/inflammatory cues or cell-autonomous stress cues dominate transcriptional outputs.

### 4.6. Limitations and Next Steps: Hormone/Cytokine Complementation, Co-Culture, and Joint FPN/Ferritin/ROS Readouts

Collectively, iron status reallocates hepatic immune–metabolic signaling; several candidates (*FGF21*, *SAA2/3*, *ETNPPL*, *ITIH4*, *RNF125*, *TFR1*) track distinct dose windows and context (in vivo vs. in vitro); and mechanistically, systemic signals likely shape in vivo expression while metal/ROS programs predominate in vitro. These conclusions are associative; protein-level validation and causality remain to be established.

A key limitation is that our validation is restricted to the mRNA level. Because transcript–protein concordance can be context-dependent, we refrain from inferring protein abundance or activity. Future studies will prioritize ELISA (*FGF21*, *ITIH4*, *SAA2/3*) and immunoblot (*TFR1*) in liver and hepatocytes to determine whether the observed mRNA patterns translate into protein changes.

Our functional annotations rely on GO and KEGG over-representation analyses, which indicate statistical enrichment of gene sets but do not establish pathway activation or causality. These approaches are sensitive to multiple factors, including (i) annotation bias and naming conventions (e.g., KEGG “viral infection” categories often comprise generic host immune modules—*NF-κB*/cytokine/endocytosis—and therefore do not imply clinical infection, consistent with our clinically healthy animals); (ii) database incompleteness and redundancy, whereby overlapping gene sets can yield similar signals; (iii) dependence on the chosen background gene universe, FDR thresholds, and software/version; and (iv) the fact that mRNA abundance does not necessarily reflect protein levels or pathway activity. Consequently, enrichment results should be viewed as associative and hypothesis-generating. Mechanistic validation will require orthogonal evidence, such as targeted protein assays (e.g., ELISA or immunoblot), perturbation experiments, and/or complementary readouts (e.g., phospho-signaling, metabolomics), to determine whether the enriched modules are functionally engaged in vivo.

This work is hypothesis-generating. Therefore, we did not incorporate hormone/cytokine complementation or co-culture to bridge systemic and cell-autonomous layers, nor did we jointly quantify FPN/ferritin/ROS alongside transcription. In future studies, we will (i) supplement hepatocyte assays with insulin and IL-6 or establish hepatocyte–Kupffer co-culture to test whether *FGF21/SAA* profiles converge toward in vivo; (ii) perform time-series FAC challenges with ROS and iron-handling proteins (FPN, ferritin) to verify the proposed threshold-feedback dynamics; and (iii) deploy gene perturbations (e.g., *MTF-1*, *NRF2*, *STAT3*) and in vivo validation to establish causality among iron exposure, inflammation, and metabolic. These steps, together with transparent data sharing (RNA-seq and qPCR raw values), should refine the mechanisms and enhance the reproducibility. No −80 °C tissue remained to enable protein assays during this revision; future work will prioritize targeted ELISA/IB once new samples are collected under a dedicated protocol. To directly test whether the observed mRNA changes translate into protein abundance and activity, we will conduct a follow-up study using newly collected Duroc piglet cohorts under a dedicated, ethics-approved protocol. Representative candidates will be prioritized for protein-level quantification (e.g., ELISA for *FGF21*, *ITIH4* and *SAA2/3*; immunoblot for *TFR1*), alongside selected signaling readouts (e.g., *NF-κB/PI3K–Akt*). These protein-level data will be disseminated in a separate manuscript.

## 5. Conclusions

This study integrated liver transcriptomics with targeted mRNA validation to characterize how dietary iron reshapes hepatic iron regulation in piglets. The key conclusions are:

1. Serum indices. Serum *HIF-1* (or *HIF-1α*, if applicable) increased with dietary iron, whereas TFR was lowest at 0 mg/kg supplemental iron, indicating iron-status–dependent endocrine/transport responses.

2. Liver transcriptome. We identified 344 differentially expressed genes (DEGs). Functional enrichment highlighted immune–metabolic signaling (including *NF-κB*, *PI3K–Akt*, and *TGF-β*), along with related pathways involved in extracellular matrix and lipid metabolism (FDR < 0.05). By integrating DEGs with WGCNA, we prioritized 14 iron-responsive candidates associated with serum hepcidin, *TFR*, and *HIF-1*—*LOC106504547*, *LOC100153899*, *LOC396684*, *ETNPPL*, *SAA2*, *SAA3*, *FGF21*, *GPR153*, *RNF125*, *AVPR1A*, *ITIH4*, *FNDC1*, *SLC44A3*, *INHBE*—and qPCR in liver showed broad concordance with RNA-seq.

3. Cell model validation. In primary porcine hepatocytes subjected to DFO→FAC treatments, *FGF21*, *RNF125*, *SAA2*, and *SAA3* mRNA displayed iron-dose–dependent patterns, consistent with a cell-autonomous metal/ROS contribution to transcriptional control.

Together, our data delineate iron-responsive hepatic genes at the mRNA level and support a layered model in which systemic endocrine/inflammatory cues dominate in vivo, while cell-intrinsic metal/ROS programs predominate in vitro. These findings nominate tractable targets (e.g., *FGF21*, *SAA2/3*, *TFR1/ITIH4*) for protein-level and functional studies to refine mechanisms of hepatic iron metabolism.

Protein-level validation in new Duroc piglet cohorts is planned and will be presented in a separate publication, complementing the present mRNA-level findings.

## Figures and Tables

**Table 1:** Diet formula and nutrition level.

Ingredient	Content (%)	Nutrient Level *	
Corn	30.0	CP (%)	19.0
Soybean meal	15.0	Ca (%)	0.80
Expanded soybean	15.0	Fe (mg/kg)	76.8
Rice	10.0	Ap (%)	0.42
Flour	6.0	Lys (%)	1.22
Glucose	4.0	Met (%)	0.39
Peruvian fish meal	3.0	Methionine + cystine (%)	0.67
Citric acid	1.50	Thr (%)	0.23
Vegetable oil	1.00	DE (MJ/kg)	13.38
Fine stone powder	0.84		
Calcium hydrogen Phosphate	0.81		
Lysine	0.18		
Fungicide	0.10		
DL-methionine	0.08		
Premix	0.49		

Note: Values indicated by an asterisk are the formulated (calculated) target values; those without an asterisk indicate analytical values determined by laboratory analysis; The premix provided the following per kilogram of diet: 12,000 IU vitamin A; 3000 IU vitamin D_3_; 50 IU vitamin E; 5 mg vitamin K_3_; 1.2 mg vitamin B_1_; 3 mg vitamin B_2_; 3 mg vitamin B_6_; 15.00 μg vitamin B_12_; 0.5 mg folic acid; 0.1 mg D-biotin; 10 mg pantothenic acid; 0.5 g choline chloride; 10 mg Cu; 30 mg Mn; 30 mg niacin; 110 mg Zn; 0.3 mg Se; and 0.2 mg I.

**Table 2:** PCR primer sequences.

Genes	Genes Accession Primer Sequence (5′–3′)	Amplification Length/bp
LOC106504547	F:CCTGAAGGGCCAGATCAAGA R:TGACATTTTCTCTGCGTTCGG	119
LOC100153899	F:AGTCACCGACCCCAGTCAA R:GCTTTATTGCTGTGCGAGGTC	204
ETNPPL	F:ATGCCAAATGAAGCACGCTC R:CGCTTGTGGCTGGGATTTTC	74
SAA2	F:ACCCCAACCACTTCAGACCT R:AGCAGTCCATCTCCTAAGCATTT	166
SAA3	F:GCTTTCCACGGGCATCATTT R:CATGTCCGAGTAGGCTCTCC	121
FGF21	F:GAAGCCCACCTGGAGATCAG R:GATCCGTACAGTCTCCCGTC	167
RNF125	F:CTCTGCTCTGCAGTTGAGGT R:CTTAGTTGAGGCGTGGAGGC	104
ITIH4	F:CATGAGGGGCAGGAAAATCCA R:GCCCTTCTCCTGGGTGCTTC	120
FNDC1	F:GGCATCCGAGTGGACAAAGA R:ACAATGGTTCGACCGTCTCC	160
INHBE	F:GGCTACACTTGAGCAGTCGT R:GGACCGAGGAGTAGACAGGT	197

Abbreviations: LOC106504547, serpin A3-8; LOC100153899, serpin A3-8; ETNPPL, ethanolamine-phosphate phospho-lyase; SAA2, serum amyloid A2; SAA3, serum amyloid A3; RNF125, ring finger protein 125; ITIH4, inter-alpha-trypsin inhibitor heavy chain 4; FNDC1, fibronectin type III domain containing; INHBE, inhibin subunit beta E.

**Table 3:** Effect of different dietary iron levels on growth performance of Duroc piglets.

Items ^1^	A (0 mg/kg)	B (100 mg/kg)	C (200 mg/kg)	*p*-Value
IBW (kg)	13.64 ± 0.54	13.47 ± 0.51	13.59 ± 0.70	0.97
FBW (kg)	27.26 ± 1.04	27.05 ± 0.69	26.03 ± 1.15	0.65
ADG (g/day)	425.44 ± 23.00	424.48 ± 14.49	388.8 ± 25.00	0.41
ADFI (g/day)	921.19 ± 39.68	917.99 ± 25.83	860.56 ± 40.06	0.84
F/G	2.19 ± 0.10	2.16 ± 0.06	2.20 ± 0.11	0.43

Note: ^1^ *n* = 6, per treatment group (mean and SEM); IBW, initial body weight; FBW, final body weight; ADG, average daily gain; ADFI, average daily feed intake; F/G, feed-to-gain ratio.

**Table 4:** Effect of different iron levels on serum parameters in the piglets.

Items ^1^	A (0 mg/kg) ^2^	B (100 mg/kg)	C (200 mg/kg)	*p*-Value
EPO content (mIU/mL)	4.49 ± 0.14^a^	4.90 ± 0.22^ab^	5.14 ± 0.17^b^	0.058
HB content (μg/mL)	66.76 ± 2.30	68.64 ± 1.12	64.68 ± 2.36	0.4
HIF-1 content (pg/mL)	343.39 ± 3.15^a^	374.22 ± 12.67^b^	414.28 ± 8.94^c^	<0.0001
Serum iron level (mg/L)	13.06 ± 0.76^a^	10.73 ± 0.48^b^	11.75 ± 0.76^ab^	0.07
TFR content (nmol/L)	16.27 ± 0.28^a^	19.64 ± 1.12^b^	19.54 ± 0.56^b^	0.008
HEPC content (ng/mL)	1182.83 ± 42.57	1208 ± 66.32	1227.33 ± 56.40	0.85
TIBC content (μHBmol/L)	147.83 ± 8.72	149.36 ± 7.66	149.36 ± 6.41	0.26
GSH-PX viability (enzyme viability units)	992.45 ± 118.91	904.15 ± 86.84	904.15 ± 99.84	0.66
MDA content (nmol/mL)	1.75 ± 0.48	1.61 ± 0.12	1.61 ± 0.17	0.9

Note: ^1^ *n* = 6, per treatment group (mean and SEM); ^2^: Different lowercase letters (^a^, ^b^, ^c^) indicate *p* < 0.05; no letter indicates *p* > 0.05.

**Table 5:** Effect of different iron levels on the iron content of piglet viscera.

Items ^1^	A (0 mg/kg) ^2^	B (100 mg/kg)	C (200 mg/kg)	*p*-Value
spleen (mg/kg)	198.78 ± 44.22	135.84 ± 16.42	180.70 ± 36.95	0.21
liver (mg/kg)	79.91 ± 11.39	95.85 ± 16.48	108.26 ± 5.15	0.43
lungs (mg/kg)	77.87 ± 11.39	72.59 ± 16.48	76.14 ± 5.15	0.68
heart (mg/kg)	32.25 ± 4.81^a^	44.28 ± 1.70^b^	48.57 ± 2.91^b^	0.012
gallbladder (mg/kg)	37.09 ± 4.03	45.61 ± 2.73	40.80 ± 9.23	0.43
duodenum (mg/kg)	32.25 ± 1.27	24.81 ± 6.42	39.45 ± 5.52	0.13
empty stomach (mg/kg)	22.17 ± 2.99	19.38 ± 1.18	27.05 ± 6.08	0.42
ileum (mg/kg)	19.49 ± 4.23	25.26 ± 8.39	31.56 ± 5.28	0.42
appendix (mg/kg)	44.54 ± 9.04	35.89 ± 6.67	44.29 ± 6.53	0.6

Note: ^1^ *n* = 6, per treatment group (mean and SEM); ^2^ Different lowercase letters (^a^, ^b^) indicate *p* < 0.05; no letter indicates *p* > 0.05.

**Table 6:** Sequencing data statistics and comparison results.

Samples	Total Reads	Clean Reads	Clean Bases	GC Content	% ≥ Q30	Mapped Reads
DGZA1	79,413,318	39,706,659	11,869,181,074	50.23%	93.59%	76,416,860 (96.23%)
DGZA2	43,121,842	21,560,921	6,454,203,722	49.27%	93.94%	41,822,247 (96.99%)
DGZA3	40,057,702	20,028,851	5,995,758,474	49.01%	93.44%	38,763,058 (96.77%)
DGZB2	42,296,658	21,148,329	6,331,325,796	49.27%	94.67%	41,102,259 (97.18%)
DGZB4	39,758,812	19,879,406	5,937,886,126	50.75%	93.20%	38,221,228 (96.13%)
DGZB5	73,804,472	36,902,236	11,043,034,970	49.66%	93.69%	70,894,004 (96.06%)
DGZC1	40,283,306	20,141,653	6,029,912,910	50.16%	94.08%	38,966,953 (96.73%)
DGZC2	38,679,518	19,339,759	5,787,524,674	49.62%	94.28%	37,324,752 (96.50%)
DGZC7	43,304,726	21,652,363	6,476,755,962	50.20%	94.73%	41,881,315 (96.71%)

Note: DGZA1, DGA2, and DGA3 represent samples of the low-iron group; DGZB2, GGZB4, and DGZB5 represent samples of the control group; and DGZC1, DGZC2, and DGZC7 represent samples of the high-iron group.

## Data Availability

The original data presented in the study are openly at PRJNA1230381 http://www.ncbi.nlm.nih.gov/bioproject/1230381 (accessed on 30 July 2025).

## Supplementary Materials

The following supporting information can be downloaded at: https://www.mdpi.com/article/10.3390/cimb47100843/s1.
