# Supplementary material for: Joint Transcriptomic Analysis of the Effect of Iron Concentration on Piglet Liver and Functional Validation of Iron Regulatory Genes
Source: Curr Issues Mol Biol. 2025 Oct 14;47(10):843. doi: 10.3390/cimb47100843 (PMC12562911; doi:10.3390/cimb47100843)
Supplement: Supplementary file 1 [file cimb-47-00843-s001.zip › Supporting Document - Table S2.pdf]

Table S2.Differential expression of core genes.

Gene	low iron group FPKM	control subjects 100mg/kgFPKM	High-speed rail group 200mg/kgFPKM	regulated
LOC106504547	944.89±577.85	2213.44±925.97	2584.88±1631.65	up
LOC100153899	741. 13±499.96	1874.94±770.22	2469.54±1733.60	up
LOC396684	384.66±125.36	811.35±427.57	1376.54±1052.03	up
ETNPPL	53.25±23.52	48.88±27.17	13.63±5.35	down
SA A 2	185.76±84.72	605.70±292.45	1133.59±624.29	up
SA A 3	33.85±10.87	90.42±14.34	162. 14±77.74	up
FGF21	2.67±3.32	2.60±1.39	14. 17±12.43	up
GPR153	0.55±0.20	1.43±0.63		up
RNF125	44.00±29.12	89.32±40.52		up
A VPR1A	3.09±1.85	8.63±6.02		up
ITIH4	954.30±60.16		2614.32±1260.30	up
FNDC1	1.89±1.07		4.35±1.83	up
SLC44A 3	3.61±2.25		8.22±4.95	up
INHBE	8.57±3.04		18.52±15.28	up
