# Supplementary material for: Joint Transcriptomic Analysis of the Effect of Iron Concentration on Piglet Liver and Functional Validation of Iron Regulatory Genes
Source: Curr Issues Mol Biol. 2025 Oct 14;47(10):843. doi: 10.3390/cimb47100843 (PMC12562911; doi:10.3390/cimb47100843)
Supplement: Supplementary file 1 [file cimb-47-00843-s001.zip › Supporting documents-Table S1 All_gene_module_kME.pdf]

#ID	gene_name	KME	Module	DGZA1	DGZA2	DGZA3	DGZB2	DGZB4	DGZB5	DGZC1	DGZC2	DGZC7
gene13474	CDKN1A	0.919976990941954	blue	18.648668	4.256383	7.911635	7.056746	5.945493	6.180579	9.066675	6.726964	5.210659
gene26961	TMSB4X	0.968042368611395	turquoise	529.783123	264.466156	307.977888	362.738037	299.237358	504.662726	223.012955	311.039112	1026.88026
gene7777	TAGLN2	0.959326129758686	blue	186.572654	51.875449	67.832573	58.079116	64.176345	73.870438	74.380575	64.865609	71.671832
gene14583	SPATA7	0.921123969266222	red	2.012301	0.510121	0.775837	1.740394	0.443219	1.532346	1.568506	3.197633	1.411612
gene13273	LTB	0.908808502137698	turquoise	0.854459	0.453215	1.281213	0.787928	0.802025	1.414546	1.540154	1.433706	3.780226
gene25739	SRXN1	0.9295852453635	blue	14.366511	3.296218	5.362148	4.13292	4.035546	4.346502	2.038357	3.260465	3.276494
Sus_scrofa_newGene_5800	--	0.954950081534985	brown	0.567521	3.930202	1.334458	0.576754	0.85131	0.68043	1.682603	1.082459	0.311029
gene12793	PPP1R3G	0.831514974059182	blue	3.722298	1.824924	1.165858	0.949413	1.082629	0.531097	0.683259	1.075716	0.409028
gene23164	MSMB	0.897505724484981	turquoise	3.857997	6.971455	6.697062	3.320852	4.669031	6.519463	3.963406	4.119049	14.328221
gene18184	LOC100625049	0.911556997607128	brown	10.703294	105.722775	31.407757	50.539625	26.956009	28.140007	65.945429	13.321935	50.308268
gene8298	OVGP1	0.774127499781303	brown	0.584451	3.29709	1.334488	0.96368	1.825444	2.007501	0.845958	1.301219	1.637589
gene19438	ATP5G1	0.934199653366763	blue	105.626395	25.102213	22.741536	28.651343	28.287608	49.427576	29.480898	45.350201	55.968656
gene2496	MRPL23	0.984386089587917	turquoise	24.346872	21.294441	11.053525	20.507283	16.965361	25.929159	19.939585	19.21115	59.166529
Sus_scrofa_newGene_15850	--	0.741894728473303	brown	0.862237	2.736524	0.370398	1.731063	0.983381	1.458494	1.088992	1.522012	0.97823
gene2819	FADS1	0.765114593714085	green	20.204926	56.610012	69.077919	28.929325	32.524361	15.028262	109.064964	17.947481	13.605847
gene24859	RETREG1	0.724591988838561	green	4.715007	2.503127	2.941062	4.076837	3.57856	1.014585	7.961623	2.011986	1.857196
gene3973	UBL5	0.941997283729723	turquoise	17.154868	8.954622	5.741744	8.881862	9.797841	15.081386	6.464988	11.125904	30.297546
gene12585	LOC100737897	0.960611731337738	green	4.628402	16.970323	15.706839	22.638832	20.32539	13.466348	39.726838	14.058623	11.575827
gene23075	PPIF	0.981359507550062	blue	232.338994	25.283488	38.621891	84.40845	45.21581	68.736576	46.101089	111.061347	45.62846
gene19916	TXNDC17	0.91208805642565	turquoise	23.393753	16.034992	16.303307	14.702627	14.494094	20.055082	12.162772	32.894882	58.495102
gene9511	MAGOHB	0.877027856864248	turquoise	2.89889	1.560661	3.350491	2.856349	1.492796	2.767707	1.195989	2.202314	5.837493
gene14034	GZMH	0.979806863321663	turquoise	1.126696	1.507597	0.968888	1.071965	0.608709	1.779461	1.061466	1.012775	4.276523
gene9133	MYL6	0.845838020739601	turquoise	198.99084	76.468514	74.707935	74.682536	81.261662	121.147779	75.305955	93.648665	243.683385
gene682	COL12A1	0.798246145543098	blue	5.478136	1.51096	1.039853	4.25279	2.773601	2.185431	1.474126	2.179091	1.973822
gene6336	LOC100521659	0.776462620802816	turquoise	1.305077	1.446902	0.79183	1.730488	2.063381	2.98551	0.77629	0.868781	3.583636
gene12583	CYP4A24	0.88244176893933	green	5.918351	26.433296	28.134092	20.992834	34.172226	21.661081	69.194267	17.599062	13.300155
gene23454	USMG5	0.899422499838592	turquoise	31.853424	9.365981	14.17319	12.577646	14.250917	19.718462	6.265017	18.344292	50.489761
gene18922	UBALD2	0.983764325242736	blue	34.994206	6.97321	10.668031	9.162008	9.751138	12.426466	7.903831	13.417427	11.325208
gene22939	DNAJC12	0.918021458061835	blue	82.478167	34.513002	24.755352	30.435787	25.371376	37.910689	16.821386	27.678825	38.527112
gene14665	CLMN	0.711671337796809	blue	6.130702	2.69243	1.511212	5.6802	2.235499	3.381893	3.259315	3.15779	1.266151
Sus_scrofa_newGene_3249	--	0.860124391842889	brown	0.69183	5.026063	1.273805	1.849857	1.281423	0.204699	1.583881	2.738458	0.324717
gene23880	SCTR	0.861993711688601	black	1.137853	1.75289	1.069906	2.757572	0.439107	0.758162	1.36921	0.222876	1.177171
gene11884	ATPIF1	0.906258611753691	turquoise	78.919128	30.351151	30.227358	47.028175	43.604801	53.46579	28.235355	46.934143	118.710564
Sus_scrofa_newGene_9866	--	0.844834184297754	brown	0	4.329161	1.908012	1.944762	1.687286	2.499354	3.312054	1.692985	1.690874
gene10722	FBXO17	0.927396106112174	blue	3.43997	1.037532	1.245757	0.805135	1.604206	1.340421	1.051595	1.860534	1.189247
Sus_scrofa_newGene_8184	--	0.858473022271307	green	0.989327	1.814031	1.580302	10.06894	2.096763	1.953955	11.074859	5.117436	2.122005
gene23234	ACTA2	0.967724667374155	blue	200.888255	12.213657	14.569747	26.860781	11.260684	52.876903	17.992638	13.900672	30.489862
gene4146	RPL36	0.979611073893453	turquoise	106.650032	67.430138	66.808136	62.162048	76.75808	99.994576	101.05529	82.377022	318.679932
gene9536	LOC100520491	0.939670025644649	turquoise	4.69459	6.21836	7.125254	9.645442	4.08632	9.668228	4.833477	4.867373	21.100649
Sus_scrofa_newGene_5115	--	0.774813729475575	turquoise	0.273498	1.7514	0.635402	1.774293	1.855171	1.571927	1.590562	0.965606	3.71187
Sus_scrofa_newGene_8092	--	0.73530404834667	brown	0.602861	3.271399	0	1.162458	1.100879	1.29148	1.217189	2.235627	0.887717
gene25043	GPX8	0.914930894998331	turquoise	2.909396	1.247992	1.604292	2.843967	1.350647	2.405001	1.040086	0.783191	5.698849
gene4463	HMGCR	0.880746802977407	green	7.48372	24.644779	7.830572	15.577455	9.77331	6.352555	36.162575	6.531019	13.987843
Sus_scrofa_newGene_1591	--	0.81116224603397	brown	1.757628	5.522776	0.441885	1.673137	0.513849	1.598423	4.12891	0.716115	1.956148
Sus_scrofa_newGene_10537	--	0.900713107390058	brown	0.119711	4.039171	0.879428	1.538168	1.816848	1.031515	0.851573	0.723408	1.603705
gene25649	CST7	0.933796183321398	turquoise	1.448053	1.517563	1.550077	2.556623	1.645197	2.134633	2.220808	0.674545	6.40169
gene295	ARG1	0.837061957340228	blue	1332.748589	334.021375	637.533669	598.180794	388.967153	517.566849	234.575668	229.925621	596.233364
Sus_scrofa_newGene_16247	--	0.826861986505261	turquoise	1.166941	1.087219	1.116363	1.84263	0.870755	0.521756	0.766061	1.553382	3.218789
gene22471	SDS	0.722687050001072	blue	51.536095	10.306522	22.813086	5.389558	10.676364	13.140086	4.765884	2.413565	32.073238

Sus_scrofa_newGene_13529	--	0.96978166169762	turquoise	77.135145	68.0057	33.639398	72.135469	63.640681	80.697928	67.565359	71.273321	183.708392
gene28138	LOC100624149	0.96236450986487	black	0	34.603476	0	31.451614	0	0	0	0	0
gene20567	FAM212A	0.924981146741284	turquoise	0.999963	0.489706	0.531929	1.279915	0.486746	0.955593	0.833697	1.101952	2.562732
gene16464	CASP1	0.977067561375247	turquoise	5.495666	7.430752	5.750967	8.26263	6.301854	11.205106	4.706353	5.099502	27.756123
gene23975	ACSL1	0.774865446471804	blue	1469.320679	186.19922	286.699729	679.683929	288.254887	1316.347045	546.403448	1090.782723	265.660643
gene3602	UBA52	0.989626758323691	turquoise	314.440798	205.429835	224.957482	238.252179	213.627921	273.789302	241.799738	251.24387	764.814297
gene26313	TMEM176B	0.709175043321221	turquoise	3.875931	0.945034	2.807114	2.146875	1.849292	5.717046	1.88387	4.307759	6.189316
gene13540	PGC	0.70008492597208	black	1.459871	3.060039	1.18774	3.337801	0.440696	3.319384	0.967149	2.151249	1.281534
gene19745	RPL23A	0.987135067015555	turquoise	255.383362	172.008133	157.843765	172.808762	193.366974	224.737656	164.596252	204.37291	598.918457
Sus_scrofa_newGene_1350	--	0.793202009881317	red	9.132262	1.802161	2.26393	4.888864	1.505473	5.136112	6.181119	10.175711	3.304421
gene12839	ELOVL2	0.903637329566125	blue	50.99628	3.227489	18.568772	15.92557	8.458073	12.455861	13.512548	31.749985	5.000418
gene25831	ACSS2	0.953891586692576	brown	15.428773	56.671199	25.766859	24.638497	22.341932	17.08867	39.280946	16.00923	19.777882
Sus_scrofa_newGene_2640	--	0.820270351102021	brown	0.443623	3.998188	1.287955	1.796494	1.420705	1.592717	1.589202	1.255716	2.983295
gene10825	CD79A	0.779368236656712	turquoise	1.392448	1.153391	2.721527	1.496106	1.649193	2.296342	3.999015	1.044582	6.030382
gene11722	MINOS1	0.851009852262216	turquoise	34.075905	11.38514	12.21874	14.171176	14.086622	20.397814	11.984264	16.738167	42.637093
gene8854	C5H12orf75	0.935754993949247	turquoise	1.538395	3.123137	1.754135	2.790671	1.940019	3.621497	2.155516	2.394691	7.036664
gene23064	KCNMA1	0.807875791085445	brown	1.533109	7.731126	5.687775	3.070206	4.999597	0.876299	5.056748	1.566733	3.016872
gene7990	S100A13	0.967208889269204	turquoise	13.044776	13.651231	10.401135	14.498465	9.327002	14.573073	14.913093	18.18967	47.326532
gene20470	CCRL2	0.953620799403827	turquoise	1.463534	1.974417	1.100501	1.607967	1.673726	1.908937	1.854838	1.262969	4.653417
gene15291	PARM1	0.803512193110596	blue	21.227592	3.441542	11.609385	3.304696	9.242114	9.396656	5.883142	4.91463	7.217678
gene6721	CGREF1	0.810887141678598	brown	1.279613	2.556487	0.988385	0.96712	0.776397	0.959214	0.781629	0.571449	1.314603
gene10876	LOC100525346	0.901307603807784	blue	5.404435	0.943461	1.44542	1.46931	0.946746	1.607193	1.043616	1.095001	2.724155
gene6478	LOC100514282	0.72721835821769	turquoise	5.731073	5.419612	3.049325	7.329336	3.933436	5.573727	2.003874	9.940868	12.133541
gene5947	MCT7	0.879088353190122	turquoise	1.769782	1.536914	1.212801	2.577245	1.455424	1.664609	2.743181	1.623008	4.933382
gene24886	LOC100525311	0.733415133165787	blue	8.534966	2.462128	2.721658	3.297259	0	2.500415	2.921762	1.926187	6.413615
gene890	ONECUT1	0.704974122735605	brown	6.531817	24.823866	8.039118	19.958614	8.837415	8.662963	23.690915	14.550938	5.133922
gene27019	SCML1	0.91560116635515	brown	1.082076	4.764453	1.707228	2.343514	1.544751	1.492224	1.901093	2.434306	1.108942
gene14033	GZMB	0.849186738766062	turquoise	0.22581	1.436434	0.773388	0.827117	0.750605	0.488893	1.126066	0.521335	3.252365
gene25950	ADA	0.915756933029106	turquoise	1.072243	1.291967	1.541161	1.06737	1.210258	1.573205	1.199954	2.1067	4.075642
gene17424	CENPF	0.819609334200753	brown	0.936635	3.52798	0.850129	1.560147	0.941375	1.468131	0.81019	1.018049	2.010444
gene10295	LOC100739663	0.753871649570254	turquoise	0.950124	21.522924	0.582208	0	4.530421	12.874072	9.618688	0	31.185291
gene26154	ATP5E	0.985039053375754	turquoise	114.063039	66.301026	75.443525	58.36251	73.630447	107.083667	60.356127	90.06776	305.967461
Sus_scrofa_newGene_1630	--	0.90144061897561	blue	4.876483	2.063083	1.745081	1.546551	1.230198	2.216823	1.964874	1.25154	1.738328
gene4289	C2H19orf24	0.802444522298158	blue	5.283451	0.814736	2.033683	1.418741	3.197098	2.495997	3.129743	3.04288	1.752548
gene20135	TRIM16	0.833655550608814	blue	4.670657	2.300534	0.983604	0.480989	0.794245	0.581922	0.899793	2.097624	1.616324
gene2469	RPLP2	0.986472187828438	turquoise	218.841278	111.343536	114.843048	137.116089	137.710495	187.434525	113.761864	155.284836	525.170532
gene11615	ANGPTL7	0.918707352175971	brown	0.07158	3.544026	1.780245	1.885131	1.37519	0.403072	1.607109	0.900602	1.236568
gene2515	PHLDA2	0.953144350975847	blue	6.917321	0.691439	0	0.481337	0.525225	0.501529	1.084559	3.013473	0.252126
gene13849	PRC1	0.866553283419545	blue	8.115604	1.755665	2.684857	5.242899	2.544941	4.39394	2.059388	6.157866	3.167591
gene16278	AAMDC	0.969878200145443	turquoise	11.410199	6.712386	10.385703	8.673068	9.579991	14.591623	9.404366	12.556866	31.632547
gene12636	PLK3	0.854588890432819	blue	2.620083	1.032692	0.81889	0.820697	0.710334	0.637501	1.490745	1.358253	0.496667
gene20820	CIDEC	0.92452801456008	blue	17.54589	2.623809	3.34834	7.028482	3.892704	10.557708	7.100571	8.65049	7.27573
gene11588	SLC25A33	0.888149476125359	blue	10.810491	0.377573	1.532074	2.346339	2.02052	2.564303	1.322863	8.219957	1.896292
gene12584	LOC110255311	0.915162638130545	green	3.897503	18.756281	18.992154	16.138635	20.534233	15.440507	43.767948	14.486786	10.895958
gene21027	PAQR9	0.756220669226004	red	3.658208	1.810317	3.151567	8.297029	2.438995	3.699642	2.684356	7.190308	1.775004
gene2473	POLR2L	0.960881915382406	turquoise	3.4447	2.102896	2.336154	1.779926	2.731641	3.236822	2.09809	2.107675	6.844544
gene2228	RPL35	0.995262823451712	turquoise	264.801971	208.346024	205.969086	213.622696	197.298782	299.259766	217.329071	253.137207	765.528564
gene7514	CYP7A1	0.770707672245722	brown	0.123825	65.350906	55.927216	9.869325	8.059879	11.198966	48.428795	3.288669	13.14502
gene12418	PGM1	0.946439850947833	blue	97.439106	26.145433	28.046278	25.348148	25.744656	23.992634	32.307777	25.380238	25.148783
gene17485	G0S2	0.87101736609697	red	22.317926	0.452694	3.067696	13.319773	14.336189	10.522968	11.161989	38.355142	5.894448

gene15359	TMEM154	0.931197761999236	turquoise	1.31391	1.586842	0.657555	1.464122	0.897124	1.098093	1.485279	0.831316	3.749453
gene9518	LOC100523789	0.722483500410465	turquoise	1.313216	1.855702	3.11898	0.914048	1.610414	1.550374	2.630373	1.524138	4.579152
Sus_scrofa_newGene_7334	--	0.805628757010009	black	1.803892	3.971739	1.028864	2.731129	1.116901	1.272468	0.190932	0.813809	0.573448
gene5735	LITAF	0.937110162393408	blue	63.598895	13.943395	26.171621	21.349514	21.175368	33.476501	17.994118	28.6264	31.927591
gene7014	SLC45A4	0.968998571556254	blue	6.96329	2.409381	2.81431	2.625821	2.110378	2.892419	2.370136	2.579811	2.149398
Sus_scrofa_newGene_14861	--	0.761605995453158	red	1.772192	0.969031	0.427401	1.455892	1.033276	1.137965	1.142189	5.13098	3.599528
gene10886	APOC4	0.965403821772923	turquoise	136.451782	179.56398	190.905411	164.787262	162.241837	200.191956	166.336334	185.833237	554.927063
gene10294	MT1D	0.944003496587076	turquoise	655.736041	961.303299	1219.586457	610.330478	227.242354	1318.185305	1055.890875	589.831297	3753.713567
gene22249	GALNT7	0.961652648726342	blue	5.46251	1.71123	1.831657	1.790209	1.344567	1.968419	1.26572	1.610534	1.786349
gene26109	PFDN4	0.966357160345018	turquoise	2.526974	1.396266	0.547404	1.736461	1.39337	2.401858	1.890368	1.780791	5.85817
gene10788	CYP2B22	0.750099511510472	brown	6.529803	20.77663	12.562072	0.477905	11.175156	6.453363	16.064703	1.4665	5.232396
gene6471	EFEMP1	0.725047199319444	blue	3.619302	1.032076	0.86994	2.582746	2.008939	2.195152	0.631012	1.291621	2.37466
gene16210	KCNE3	0.895925825225511	blue	24.900341	4.959733	10.043689	5.180352	8.355935	7.164412	4.58329	5.060104	7.662952
gene10112	WFDC1	0.837365207225937	blue	6.954566	2.101133	2.040115	1.998344	1.413323	2.931698	3.762901	1.237713	2.992939
gene8002	S100A12	0.911269085976271	turquoise	1.968362	0.579913	0	0.989299	0	1.558348	0.143817	1.983468	4.642817
gene19705	EVI2A	0.918106270226198	turquoise	1.475751	1.047529	1.137899	1.472971	0.546145	1.462517	0.372649	0.932945	3.016051
gene19967	BCL6B	0.984089715634205	blue	7.506517	0.812252	1.154436	2.007071	1.774006	1.558098	1.415351	3.009341	1.698534
gene23340	PGAM1	0.961892348559963	blue	153.954651	44.911434	41.49017	56.030041	45.52636	51.73761	60.897549	48.004467	52.051163
gene222	ABRACL	0.879041266371299	turquoise	2.36038	1.002828	1.601667	1.089847	0.971472	2.859525	1.953437	2.73561	4.92592
gene776	RAB27B	0.906321959254253	blue	6.105979	1.065533	2.110279	2.246623	2.83858	1.785986	2.878943	3.057565	1.814943
gene9587	C5H12orf57	0.987213938021506	turquoise	9.80706	7.470888	6.49284	7.367767	8.818192	10.63444	8.297725	8.238893	24.601715
Sus_scrofa_newGene_5253	--	0.923694598243884	brown	1.457746	16.19256	2.260483	2.249979	4.969617	4.795318	3.291178	0.670801	2.963859
Sus_scrofa_newGene_7927	--	0.756706909929493	brown	0.380855	3.897459	1.262856	0.965593	0.900129	1.290372	0.656897	1.980681	2.605159
gene16264	CAPN5	0.70135496862922	black	1.165207	2.561243	1.046337	5.371272	1.408127	2.965041	1.860377	2.915418	3.583036
gene16671	HSPA8	0.977457055253267	blue	518.993591	131.971817	143.937042	178.607697	119.105629	272.354614	139.695694	227.924698	189.335037
Sus_scrofa_newGene_6341	--	0.733107947784007	turquoise	0.50026	1.769595	1.05243	3.682639	0.880875	2.037317	3.237791	2.143277	5.54403
gene22076	CKS2	0.724425868410645	blue	12.085117	3.728806	4.703941	2.939487	3.7375	5.278734	1.756038	4.078337	9.897796
gene19092	CD79B	0.935343196816103	turquoise	1.046925	0.887106	0.45419	0.299227	0.86412	0.801859	1.218008	0.423693	3.557278
gene2603	C2H11orf86	0.975316830762784	blue	8.211124	0	0.162238	0	0.31068	0.944042	0.69635	0.850406	0.896626
gene17447	BATF3	0.937931261330681	turquoise	1.46123	1.260917	1.868008	0.813657	1.712083	1.530499	1.373699	1.238278	5.116237
gene13625	PLA2G7	0.896793518692544	blue	28.955928	5.220885	7.614297	15.70711	6.774002	15.117036	5.079689	18.742237	4.979685
gene10127	MLYCD	0.917448817493213	blue	19.80653	4.746695	6.084863	12.756063	5.63435	10.232338	7.086809	11.857685	6.313443
gene4138	NDUFA11	0.812811720112068	turquoise	41.863735	15.654463	16.015469	14.92298	21.695911	20.676289	17.855558	18.167097	50.084652
gene7603	XCL1	0.927914764772063	turquoise	1.055591	1.629173	0	1.003287	0.681626	1.520828	0.441554	1.010148	3.751239
Sus_scrofa_newGene_3620	--	0.705595946266975	red	4.331698	1.23629	1.288861	8.549829	3.939142	4.595752	2.99862	6.253974	2.880634
gene12722	CTPS1	0.947121791638066	blue	2.968785	0.498543	0.559657	1.012218	0.435274	1.666041	0.461982	0.993868	0.579311
gene14420	RDH11	0.92055095606222	blue	439.137602	76.366826	140.759444	100.730207	106.77709	257.714183	82.184289	253.099323	133.296838
gene14554	SLIRP	0.904495845921204	turquoise	14.935291	3.834554	9.465074	4.565388	4.291025	9.722097	3.264074	7.151774	25.740742
gene23152	C14H10orf128	0.938810999225112	turquoise	1.333478	1.344994	1.615132	1.291623	1.496643	2.266393	1.88539	1.04194	4.328516
gene14113	MRPL52	0.905270956163255	turquoise	8.373945	2.535762	3.874207	4.48915	3.566129	6.3481	3.322286	6.936282	14.013714
gene27869	LOC100518170	0.730818069212537	turquoise	1.532877	0.068808	1.075499	0.557901	1.62814	1.545933	0.479096	0.356532	2.56367
gene26185	RPS21	0.990624235025917	turquoise	129.277084	66.647903	72.052795	75.77282	85.675148	120.336662	80.892426	97.969917	460.736877
Sus_scrofa_newGene_2712	--	0.726009123012476	brown	4.150915	11.205621	5.024997	2.433944	9.399986	2.239154	4.760066	3.386597	1.976177
Sus_scrofa_newGene_12616	--	0.844778335576798	green	0.425847	1.207161	0.879724	2.151437	0.986247	0.80049	2.055504	0.924669	0.649465
gene4935	SRA1	0.907451352669302	blue	5.974695	0.790941	1.054189	0.197434	1.915677	0.492409	0.751388	2.231764	1.321651
gene11572	ERRFI1	0.779643655284129	brown	27.576267	87.477737	33.373791	35.451855	36.875961	16.80106	44.376747	69.229279	29.647661
gene13057	LOC110261659	0.9913494371833	blue	8.738103	0.627384	0.430214	1.764423	0.740461	1.52983	0.283556	2.954834	0.974592
gene24961	RPL37	0.980065716902416	turquoise	227.007416	79.509575	78.016342	83.386658	130.462662	160.725266	79.016617	135.827118	686.490906
gene9234	AVPR1A	0.778531634732199	red	5.188636	1.728605	2.34066	13.990627	2.126504	9.785686	3.594921	11.22031	2.607737
gene16324	LOC100519675	0.95092345685574	turquoise	12.678467	3.66682	4.711962	4.346217	3.846996	9.152378	3.175061	9.422934	27.48258

gene14655	LOC396684	0.954883788057472	red	512.022156	261.394318	380.566559	1285.006958	453.894958	695.150818	962.6698	2572.574707	594.388062
gene4183	EBI3	0.881271243407284	turquoise	1.065449	2.017317	0.90261	1.20732	2.367491	2.256986	2.183178	1.963762	5.337306
gene13490	CCDC167	0.941460046692389	turquoise	7.785762	5.726253	6.524211	7.930473	8.853996	9.562563	9.036426	10.861875	22.922165
gene15230	LOC100624700	0.723288897354117	blue	12.225589	7.230995	3.314338	1.805534	5.999264	5.203811	6.610865	9.381202	3.63723
gene23602	DMBT1	0.775628516554954	green	4.295598	2.272885	2.36781	1.827617	2.491321	3.446738	12.713217	1.352589	1.896979
gene8654	TSPO	0.968892197084666	turquoise	4.038003	2.748165	3.429116	4.569657	2.910756	4.383124	2.303066	4.009459	10.248983
Sus_scrofa_newGene_11202	--	0.788774968055176	green	0.239753	1.657231	0.276149	1.873378	0.951681	0.414671	1.907043	1.219706	0.861807
gene11838	CD52	0.956171950558197	turquoise	3.354256	2.166186	1.984445	1.82107	3.801687	3.554638	5.187172	3.954582	19.958599
Sus_scrofa_newGene_9020	--	0.987206966268778	turquoise	23.347182	12.420686	14.298877	16.378758	17.21797	21.195634	14.339381	16.847438	58.846012
gene1411	RPL36AL	0.986604887783531	turquoise	77.285339	53.694069	60.918728	50.050354	55.423477	85.307869	38.627525	66.220688	218.033096
gene8693	PMM1	0.870347773085199	blue	105.073776	3.800567	22.055719	30.656973	27.82819	67.744171	19.03075	73.251625	23.708845
gene24545	CXCR2	0.976959965429214	turquoise	2.847297	1.200403	1.922506	1.873301	1.541074	2.597368	2.494782	1.785598	7.738338
gene11683	FAM131C	0.977087223716748	blue	8.88797	0.447081	1.254878	2.330463	1.335527	3.6493	1.696649	3.303462	2.772392
gene19004	RPL38	0.972830205433166	turquoise	67.177044	22.384302	20.347949	20.521581	40.289723	51.228126	22.627377	42.741962	187.593258
gene11917	FABP3	0.812070600017911	blue	7.598713	1.798314	0.54974	1.591069	1.064322	3.438972	2.658468	1.047795	4.488188
gene18799	PYCR1	0.94359653297942	red	6.371353	4.170035	3.886069	6.451293	4.530055	5.828913	5.061847	13.227706	2.970169
gene19696	COPRS	0.785724131989137	turquoise	1.280125	2.022212	1.304723	0.462978	1.077689	0.84939	0.97922	1.282857	3.046083
Sus_scrofa_newGene_7798	--	0.915959945031955	red	0.634367	0.121639	0.695904	1.702442	0.237343	1.985404	0.628237	2.769301	0.748282
gene28142	LOC110257905	0.990759388323335	black	0	25.15662	0	31.288309	0	0	0	0	0
gene5853	TNFRSF12A	0.983644342704773	blue	11.771169	0.473649	0.383351	0.751373	0.31324	1.614369	1.632288	1.937275	1.329924
gene11275	LOC100511639	0.908186522725326	turquoise	6.006021	10.191659	2.287173	4.610396	2.438655	13.404616	2.796154	2.541919	23.235753
gene13590	POLH	0.840357332687249	brown	2.427641	7.685341	3.795711	2.385589	3.147314	2.321484	7.113332	2.524739	3.415126
Sus_scrofa_newGene_13145	--	0.81310770715717	brown	0.709071	2.579723	0.480708	0.912581	0.734603	0.646896	0.789205	1.588192	1.138864
gene28841	EXOC3L4	0.832223490865801	brown	1.417555	5.165636	1.861171	1.833615	1.730149	2.096865	2.08104	3.432402	1.247526
gene6238	GNLY	0.72623834962008	turquoise	8.27854	13.383004	10.046405	10.798788	9.024369	11.944293	24.004593	9.335398	29.469158
Sus_scrofa_newGene_14137	--	0.830129139014477	brown	0.303071	2.687587	0.9228	1.283637	1.024783	0.545816	2.055701	1.748896	0.675838
gene1346	RPLP1	0.997414647060061	turquoise	570.371765	364.469147	332.277618	437.170807	343.092163	546.552368	363.261932	404.625824	1529.355591
gene4158	PLIN3	0.893777741106822	blue	61.119872	8.305151	9.849738	25.685695	12.45625	35.084919	23.907198	42.688731	22.814871
gene20238	COL6A5	0.809583276454398	black	0.491261	2.440382	1.502188	12.370287	3.104344	2.953789	1.772228	1.998404	2.179769
gene16568	APOA4	0.952051329024871	blue	4899.980957	65.572945	612.370544	1155.450806	459.032867	2509.590576	564.66449	2108.822998	891.755615
gene21457	RPL35A	0.993613808173613	turquoise	108.323621	71.799243	65.328279	78.189331	76.004609	111.228905	65.37143	87.795036	439.137686
Sus_scrofa_newGene_4377	--	0.869643992872899	green	0.496916	4.070601	2.05872	2.316182	2.25081	1.741889	6.483991	2.072904	3.915329
gene24771	PER2	0.936810526089008	brown	2.178593	8.858339	4.808542	3.849094	4.339848	2.239511	3.351371	2.607269	2.999506
gene12975	LOC110261477	0.944713700434716	turquoise	2.595151	1.943436	0.800463	2.11052	1.652913	2.370476	3.909278	2.930829	10.61021
gene14723	ATP5I	0.728339456756188	turquoise	39.304016	4.527093	8.13033	6.614748	9.34982	16.609325	5.013585	10.381966	38.996494
gene5641	IGSF6	0.953079755565205	turquoise	2.892998	2.872595	3.118164	4.054453	2.128952	3.794005	1.810851	3.262491	8.406504
gene7382	FABP5	0.801045370689427	turquoise	3.23921	1.053987	1.273525	4.182402	1.458963	3.450325	2.531891	3.355091	6.057577
gene17345	TOR3A	0.819121833120755	blue	4.526778	1.402953	1.140042	2.023723	1.286195	2.429686	1.099814	4.270929	1.421923
gene23229	LOC100157486	0.939801244847834	red	2.080034	0.291231	0.847522	3.05741	0.373604	2.884314	1.733763	4.317924	1.440556
gene19619	ACACA	0.90090132548088	brown	5.322692	20.317502	8.940078	12.314193	9.172174	5.856774	8.986047	4.141913	4.912495
gene3645	BST2	0.943196611425477	turquoise	32.222248	36.566566	24.186878	29.256683	24.607693	58.257198	19.483929	25.820164	92.380867
gene8341	LOC110260351	0.767708005864565	green	2.208938	4.976774	2.937022	2.843184	5.421128	1.068666	7.419234	3.419071	3.689246
Sus_scrofa_newGene_7732	--	0.806834519408476	green	1.234908	1.057035	0	1.916585	1.014667	1.492175	3.295214	1.150161	0.638867
gene9741	NELL2	0.909837515881674	blue	13.36443	2.545963	2.59566	7.099893	1.583524	1.920022	3.916958	4.561183	2.718886
gene26788	UPP1	0.874302022946939	blue	4.822617	0.88747	0.407207	0.47527	1.078563	0.460595	0.41456	1.135603	2.283333
gene18819	OXLD1	0.8811102777278	turquoise	2.117547	3.869496	1.237129	1.532428	2.118017	2.799091	0.843906	2.972563	7.089506
gene28136	EIF1AY	0.979420676220649	black	0	23.435595	0	24.654756	0	0	0	0.037824	0
gene14424	RAD51B	0.704464547641839	turquoise	0.567916	0.243234	1.599177	1.300926	1.287013	1.255309	1.101778	1.168973	2.507306
gene8210	PTGFRN	0.715479850989277	green	2.996563	2.022299	4.616513	3.055025	6.790654	3.42752	9.774526	2.955317	3.397464
gene14641	ISG12(A)	0.945251504506162	turquoise	121.693657	86.578926	68.332642	128.83017	55.629295	150.777908	37.588074	85.848763	284.82074

gene4487	TBCA	0.969221181005208	turquoise	28.260241	18.194475	20.585417	20.686329	23.463343	25.172323	14.092675	25.559158	65.177872
gene19632	LOC110256043	0.912891323419564	turquoise	14.303159	12.043134	8.004717	20.847707	5.942109	19.139054	10.307057	10.764233	35.412058
gene24797	COPS9	0.993631246913567	turquoise	15.648714	11.085647	8.7782	11.420097	8.580459	14.435779	9.80171	13.751092	48.978954
gene6000	HBM	0.791760721044782	turquoise	3.776726	2.409388	1.552295	1.435657	3.647529	3.169739	0.270501	1.997654	6.088072
gene9651	FGF23	0.878027835581186	blue	6.330855	0.391042	0.205984	0.212767	2.102728	2.586443	0.504942	0.297673	0
gene18825	ACTG1	0.984149617081758	blue	614.522888	175.238083	131.551697	238.319504	180.977402	281.855103	221.7677	256.655518	229.298218
gene4270	LOC100524239	0.979802228248615	turquoise	1.504709	0.058409	0.176936	0.052368	0.651214	0.682222	0.36413	1.01863	16.431631
gene9487	BCL2L14	0.713204850780832	turquoise	1.299583	1.394439	0.64004	1.916434	1.212167	2.41621	0.972911	0.394427	2.681001
Sus_scrofa_newGene_14117	--	0.963370151531615	turquoise	2.695805	2.324763	2.77415	2.390426	1.385932	2.495882	1.466695	2.611598	7.195027
gene5222	BHLHA15	0.702920750305071	red	1.381703	1.275608	1.029592	0.798579	1.316627	0.748828	0.534148	3.33218	1.363463
gene15094	CTSO	0.9260917269839	brown	0.914501	4.032467	2.368433	1.548694	1.861835	1.407349	1.457063	1.150346	1.490515
gene15763	GPAT3	0.822149365084099	blue	11.228407	1.950657	1.548396	6.026675	3.054141	5.865425	3.415129	10.016352	2.412169
gene23798	CXCR4	0.777611559088545	turquoise	2.862854	1.035511	1.228161	0.780793	0.551931	1.284212	0.743894	1.857009	3.43666
Sus_scrofa_newGene_2342	--	0.918679002949043	brown	0.24295	3.688577	0.462815	1.845101	0.567645	0.863552	1.335144	0.7964	1.195435
gene4909	MZB1	0.788332701375162	turquoise	0.616326	0.499101	0.904175	1.467587	0.76537	1.580411	2.550722	1.174364	3.759275
gene4064	RPS28	0.979918455239219	turquoise	107.042292	42.470538	44.435685	44.037599	67.90649	91.51786	62.89391	82.362519	328.217953
gene27786	SH2D1A	0.890830755929466	turquoise	1.816833	2.56641	1.53712	2.100748	0.854754	1.95284	1.127089	1.143458	4.31876
gene11051	RPL13A	0.992498715946778	turquoise	314.816703	240.524994	240.957264	257.72584	227.185984	282.927107	252.633833	254.151106	729.351168
Sus_scrofa_newGene_2949	--	0.940387026578665	green	0.145385	0.79063	0.569005	1.455175	1.299549	0.653423	2.38158	0.903296	1.044034
gene7530	CHCHD7	0.831603507940841	turquoise	7.863383	3.802824	4.892423	4.687553	3.427789	4.340445	3.567485	10.178348	15.211283
gene13298	HSP70.2	0.967051971380975	blue	136.245224	32.283386	34.951851	56.017933	28.431202	56.440254	31.183683	37.114243	33.765121
Sus_scrofa_newGene_1158	--	0.96383264957346	brown	0.639603	3.768069	1.448773	1.792617	1.773828	1.113401	1.769814	0.846865	1.332284
gene9178	INHBE	0.895721427607143	red	19.849081	1.916963	3.14963	10.500642	5.064055	10.157094	15.303344	35.146858	5.100545
gene27748	RPL39	0.984439558471851	turquoise	56.056492	28.138466	28.957125	25.128965	37.409962	46.484169	30.559956	43.654987	201.09758
gene27251	UXT	0.903258714278913	turquoise	2.183233	1.689412	1.018633	1.71464	1.458199	1.366003	1.91185	2.538963	4.776073
gene27686	ACSL4	0.921447716717183	brown	6.817948	18.55974	7.589577	8.095303	4.863935	5.449723	6.01695	6.222278	6.441354
gene8956	KRT18	0.878325682370865	blue	106.50811	32.80077	27.216694	23.503637	47.938442	62.413307	40.351669	33.71381	26.478619
gene26079	PARD6B	0.923240165037673	blue	6.640831	1.865624	2.110601	3.994397	2.065105	2.117348	1.696906	3.529032	1.862426
gene16546	ZBTB16	0.865421277457965	blue	7.329265	3.228446	2.465547	3.822824	2.408864	1.639756	2.4275	2.189289	2.346549
Sus_scrofa_newGene_236	--	0.860999680701049	brown	0.668139	3.659763	1.555142	0.375688	1.982571	1.306873	1.218502	1.035182	0.666053
gene21937	CLDN14	0.909057822160327	blue	46.844154	9.828153	24.439804	13.961835	13.261855	14.324661	12.858905	24.715762	17.196854
gene13202	UBD	0.984902990576464	turquoise	8.096281	8.845399	5.964748	8.312831	7.672973	9.84763	8.723269	8.066505	27.379496
gene27631	BEX3	0.929121210678385	turquoise	1.262239	0.558191	0.592329	0.574676	0.244411	1.398663	0.470628	1.447696	3.135333
gene11533	SMIM1	0.758612757737909	turquoise	0.536327	1.783425	0.296116	1.720299	0.677584	0.933002	1.101506	0.340979	2.86955
gene25897	LOC110257391	0.782536689050256	blue	3.508097	2.403701	1.006763	1.866758	0.989152	1.149031	1.387067	1.552169	0.85242
gene9628	PLEKHG6	0.967732291430567	blue	5.492405	0.371562	0.345052	1.912935	0.861139	1.233031	1.000307	1.018637	0.763627
gene29285	LOC110258617	0.776786541329958	blue	3.903903	1.622306	1.481588	2.297309	0.360965	0.531987	1.10579	2.7409	0.506992
Sus_scrofa_newGene_4180	--	0.979631309318782	blue	14.416636	0.917197	0.812097	1.967599	1.11598	2.996874	0.682422	6.413367	1.630028
gene4065	NDUFA7	0.982826224428217	turquoise	7.698679	7.177876	4.066628	8.341841	6.370341	9.284571	5.889314	6.238399	20.856985
gene3056	PTPMT1	0.97016301494347	blue	10.671003	1.488962	1.625236	1.938674	2.446291	4.442346	3.181267	3.559116	3.076168
gene22894	LOC110256649	0.894490717334761	green	2.067591	0.166398	1.746081	2.261659	0.007107	0.045121	46.081886	3.220029	0.405986
gene13214	TRIM10	0.880516034515405	green	0.264912	1.378507	1.056479	2.192464	1.354927	0.606036	2.357804	1.04123	0.845993
gene7094	SQLE	0.883687432200863	green	14.009583	48.265741	21.294052	40.207106	25.500254	20.02882	65.791128	15.475124	34.569557
gene8457	ABCD3	0.887139813241857	blue	377.231915	34.582792	75.183086	159.647111	65.835336	234.530874	89.284002	257.898587	78.990713
gene2419	FCN1	0.975307192935813	turquoise	3.727656	1.30926	1.750214	2.150433	1.243144	2.692033	1.388947	1.89278	8.227104
Sus_scrofa_newGene_10550	--	0.784342012888943	turquoise	7.439372	10.183225	10.419522	17.943405	4.150286	7.426686	13.842091	8.758598	27.052596
gene1789	ANXA1	0.946288222984721	turquoise	24.791792	25.3799	21.15288	29.040478	18.456734	47.001617	16.340666	22.540514	74.518784
gene827	LOC100152206	0.915988424256097	red	1.859513	0.728054	1.436135	2.839599	0.90518	3.162772	1.538777	4.308549	0.732199
gene14654	LOC100153899	0.987104030340237	red	1318.082954	435.31955	469.976509	2118.097107	1012.491333	2494.237595	1764.663284	4444.545105	1199.401841
gene25093	NDUFAF2	0.782418787359548	turquoise	2.930538	2.042329	1.72431	1.913476	1.150638	2.050377	0.312248	2.898077	4.395754

gene25948	PKIG	0.838164902466332	blue	32.344233	8.584174	11.823009	10.675612	10.629677	16.300221	12.510395	8.771069	20.832325
gene10735	GMFG	0.975459068258744	turquoise	18.08293	8.048316	13.394808	17.295585	10.294561	16.030348	7.605266	15.526691	54.092872
gene10571	LOC100623157	0.939932177335005	green	0.323775	1.088374	0.618363	1.292577	1.157245	0.951479	2.39146	1.009558	1.349833
gene12962	SLC17A3	0.97754735427754	brown	0.499013	4.792939	1.280607	0.95641	1.374351	1.225305	1.694816	1.489961	1.395526
gene21320	CHRD	0.799086384237334	red	28.152736	43.214319	13.31691	35.950586	27.828154	24.913779	23.248682	76.16136	22.64878
gene11980	GJA4	0.945258327791237	turquoise	2.126847	2.582044	2.406794	3.51763	3.35498	4.948478	3.977302	3.334599	10.690909
gene13830	PEX11A	0.879757768308293	blue	22.605547	6.625453	4.569183	12.673282	7.468862	16.321593	6.743713	13.042808	6.858343
gene5578	BOLA2B	0.942780184399929	turquoise	4.739363	2.365156	2.92661	2.194133	1.904122	4.059019	2.654978	4.312797	9.357312
gene9582	LPCAT3	0.928908718872599	blue	27.653479	6.793093	5.572316	10.674414	7.028325	11.623339	15.207129	11.997606	8.742778
gene6772	FKBP1B	0.798711970388665	blue	12.043585	2.800217	4.080174	2.842134	5.380523	4.349755	3.58853	10.980973	4.3947
gene19640	LOC100516039	0.958986267884351	turquoise	2.431461	1.828869	0.828828	0.894809	0.65583	1.413322	1.339124	2.16567	6.956495
gene17449	ATF3	0.872126315452715	blue	8.392264	2.482334	1.823562	1.129161	0.404178	1.059371	3.837086	3.301527	1.552021
gene14368	FAM174B	0.953481882594226	blue	12.377794	2.560429	2.433969	3.446218	1.882495	3.440496	4.085619	2.543909	3.572601
gene4280	RPS15	0.988650657094964	turquoise	334.411163	202.052185	190.165298	248.523468	215.843872	303.812012	252.096512	214.563034	748.145752
Sus_scrofa_newGene_5290	--	0.916534364223611	brown	0.114665	6.795993	1.661707	2.303096	1.21549	1.728484	4.488575	1.698058	3.080029
gene22180	GULO	0.785585733178231	blue	114.7131	50.023757	59.878124	34.74949	59.063456	21.239003	39.992611	58.870012	28.313219
gene6742	SELENOI	0.941484982758537	blue	34.652035	10.645687	10.861415	12.359196	9.797794	12.389694	16.704321	15.236531	6.899116
gene24811	AGXT	0.820995405773552	blue	616.901855	25.306475	93.886894	65.36969	84.56057	516.119934	61.32753	217.956512	112.163498
gene5247	ATP5J2	0.922800955633772	turquoise	106.976956	46.960298	47.300793	46.243315	55.793527	65.806707	49.101145	61.41433	166.254409
Sus_scrofa_newGene_2790	--	0.868544423919255	brown	0.937011	5.412509	1.057008	0.978066	0.803484	1.289467	1.532374	1.727825	2.964771
Sus_scrofa_newGene_5470	--	0.703972961706935	turquoise	1.221816	0.420195	0.927791	2.369001	0.666829	1.759494	1.391873	1.025171	2.676084
Sus_scrofa_newGene_13484	--	0.796521225312228	brown	0.414094	2.319116	0.666813	1.665728	1.468187	0.576971	1.069709	0.883366	0.414621
gene7534	RPS20	0.992611742749955	turquoise	289.673584	181.310638	181.705032	231.749313	186.143539	283.513336	220.371933	218.883865	647.10968
gene2821	TMEM258	0.826822290362182	turquoise	15.426011	9.67316	13.341227	7.383269	19.365046	23.174559	18.955446	28.74387	46.478409
gene28139	LOC100624329	0.965248831519719	black	0	7.234237	0	6.717823	0	0	0	0.005965	0
gene12066	MFSD2A	0.907834143888826	blue	140.362503	3.365899	35.249516	56.4935	28.284456	74.26997	58.524432	84.045231	45.411608
gene5214	AIMP2	0.902337997998777	blue	4.828207	1.770341	1.589293	1.517464	1.572854	3.062302	1.13264	1.978528	2.357106
gene20380	CSRNP1	0.825637268326518	blue	3.910602	1.940007	1.774692	0.722776	1.001473	1.685346	1.517313	1.350395	1.906948
gene9394	LRMP	0.90302765572292	turquoise	2.627161	3.622935	2.531672	2.836821	2.666227	4.220053	1.874717	0.758877	7.795946
Sus_scrofa_newGene_4190	--	0.892130688357108	turquoise	2.544158	1.352532	0.695436	1.679068	1.570851	1.354295	0	1.801744	4.995988
gene4170	PLIN5	0.926849952789445	blue	28.163536	5.340381	7.864294	12.975344	8.584204	17.286916	12.690165	16.958311	9.125041
gene16575	TAGLN	0.939886648684257	blue	123.274974	12.843796	9.362271	19.674126	16.572271	43.629926	24.299989	15.38719	39.84457
gene3256	LOC102165880	0.759908026363089	green	1.541384	2.272822	2.194301	4.848124	2.203099	4.633382	9.231645	7.484802	2.57765
gene9284	LYZ	0.958145661277209	turquoise	6.308424	6.827615	4.054742	6.585656	2.271022	7.477151	3.974808	6.032298	16.334169
gene2959	LDHB	0.901909539575368	blue	4.671329	0.437541	1.530013	1.269874	0.955093	2.766491	0.638793	2.780331	1.611952
gene7471	RRS1	0.961807628537567	blue	8.186349	2.620065	1.851979	3.652939	2.083977	3.077507	1.333613	3.290426	1.914093
gene5495	TGFB1I1	0.986762164566223	blue	5.797525	0.887763	1.176752	1.758097	1.189584	1.754428	1.399791	1.533382	1.403075
gene8782	LGALS1	0.95823773088084	turquoise	14.709213	4.12836	6.189996	8.432054	5.840051	8.37645	6.268578	9.032663	31.139851
#ID		kME	Module									
gene11984	TMEM35B	0.938659041710888	turquoise	1.298828	1.962216	2.272607	1.514447	1.276452	2.015634	0.76293	1.567522	5.698255
gene18296	ALOX5AP	0.884493624689767	turquoise	10.396603	4.581703	9.339814	9.174198	4.019039	6.251352	5.524293	7.818228	19.146091
gene2527	DHCR7	0.940437584869716	green	16.110371	46.430554	41.582024	39.185372	42.028387	27.262584	95.117555	27.860619	29.515126
gene23386	SCD	0.949072660085092	brown	4.328704	21.714359	7.449487	4.693886	5.310443	2.355326	5.468836	1.444216	3.609723
gene8674	LOC110260835	0.83496898195971	brown	1.284137	2.86322	0.751289	0.8987	0.515639	0.904617	1.628965	0.952476	1.55404
gene4291	ATP5D	0.959610534683916	blue	96.942001	28.36351	22.911795	27.7328	39.944382	44.29681	37.984272	44.092274	36.912766
Sus_scrofa_newGene_14542	--	0.868518889393455	turquoise	0.714587	1.70821	0.861698	3.394827	0.522618	3.15754	0.846628	0.480066	6.205572
gene17756	LMOD1	0.910870683281373	blue	2.600379	0.506634	0.613012	1.393221	0.336075	1.058455	0.805563	0.956535	1.269545
gene23261	PPP1R3C	0.844133916474651	green	2.25825	5.359349	1.731025	15.275284	2.97684	5.307102	19.023598	11.816672	2.691458
gene13442	RPL10A	0.977763815211088	turquoise	294.732296	170.497619	167.88402	199.73147	186.176048	229.752225	176.198563	199.268003	553.987846
gene16764	ROBO4	0.905356716315798	blue	3.564987	1.201953	1.182051	1.571197	0.825001	1.116678	0.979353	0.940895	1.688288

gene20582	LSMEM2	0.918775750421462	brown	0.873668	7.685354	2.592993	3.856127	2.693478	3.499943	3.302774	1.573537	3.286834
gene12017	CSF3R	0.88338982549591	turquoise	1.801345	1.516428	0.639544	1.234326	0.748345	1.105239	1.862001	1.108567	3.485499
gene13447	FKBP5	0.882973778764865	blue	38.04922	12.937562	10.192465	21.859774	6.607492	13.119499	6.819739	8.943229	8.00142
Sus_scrofa_newGene_15477	--	0.796485206779805	black	3.110724	6.396245	3.684685	5.283872	2.610312	2.589428	1.716954	2.676815	0.791984
gene7974	RPS27	0.983973462353131	turquoise	158.408429	72.116381	73.777436	58.944678	84.006793	115.484793	83.969593	106.066479	527.709381
Sus_scrofa_newGene_14687	--	0.927903938332421	green	1.903429	2.127243	2.008248	3.285779	1.188968	1.02751	7.106776	1.421352	1.686029
Sus_scrofa_newGene_7951	--	0.706772801543845	green	0.380363	0.269155	0.702838	1.58897	0.827956	1.363829	2.04978	0.544615	1.373894
Sus_scrofa_newGene_812	--	0.724929550074145	green	0.609472	2.301883	1.513961	2.572034	1.392205	1.151652	2.478387	1.238582	0.514867
Sus_scrofa_newGene_3062	--	0.870525444223075	blue	10.828799	2.718401	3.057567	1.919796	2.841931	3.186851	1.321341	3.600885	6.119738
gene9415	LDHB	0.893391444633381	blue	665.52948	81.858078	181.521225	224.043384	140.244104	395.560108	142.765358	401.02613	341.715409
gene12587	CYP4A21	0.819449247733652	green	34.467525	77.782143	156.921616	105.56443	116.201454	59.838333	254.690445	47.427429	40.11079
gene22631	MTFP1	0.973475219488165	blue	9.566654	2.443323	2.233936	2.088982	2.349897	2.94372	1.73202	2.906382	2.813405
gene1265	PMAIP1	0.903377515567376	turquoise	2.161648	1.893005	1.079969	1.826672	1.553136	3.312739	1.985884	1.277815	4.569772
gene9623	CD27	0.811328950562572	turquoise	1.073631	1.225099	0.654296	1.092873	0.878853	1.517169	1.713939	0.425287	2.55596
gene25040	GZMA	0.967320788044714	turquoise	1.034254	2.048306	0.90069	1.304078	0.635999	2.146934	1.106637	0.572995	6.295294
gene19102	LIMD2	0.964679530520727	turquoise	3.068114	2.287913	3.075662	3.322878	2.987664	3.331888	3.773967	3.160717	10.05517
gene17907	MOB3B	0.77870572871319	green	2.886563	1.640444	2.952939	1.604363	1.658922	0.8751	7.957369	1.126223	2.445045
gene21896	GART	0.97837687644497	blue	51.854908	12.728996	15.255237	20.870914	14.868429	22.050121	16.273335	30.021542	13.944204
gene23209	MBL2	0.916252980113101	turquoise	59.12014	50.233719	23.454533	22.531479	14.366023	97.724548	25.557152	88.093628	193.485519
gene27285	CCDC120	0.897513645856863	blue	5.412482	2.335753	1.534168	2.263471	1.761128	1.567445	2.859529	2.635167	1.454544
gene25554	HAO1	0.764352686853128	brown	11.119993	121.248367	112.884722	56.942557	57.102397	44.338827	64.164007	48.279653	65.912559
gene15903	LOC110255361	0.797464874450026	black	0.994497	4.969093	1.59611	4.286699	1.561196	4.077179	2.32915	2.162654	1.994168
gene270	RPS12	0.972533878380519	turquoise	379.693665	203.246552	190.954254	233.658707	238.788757	295.956085	173.624725	288.197845	793.783447
Sus_scrofa_newGene_3032	--	0.913030548301433	green	0.355179	1.428861	1.424542	0.93532	0.997997	0.545474	3.763169	1.194588	1.503025
gene21835	ADAMTS1	0.90256000416895	blue	6.78438	2.817558	2.093634	3.843523	0.924096	2.99852	2.53543	2.731893	2.806991
gene7674	FCGR3A	0.980176168965728	turquoise	61.198538	44.366056	50.748632	48.7306	34.800585	76.950155	49.075824	35.154583	154.475399
gene13275	AIF1	0.980424918812197	turquoise	18.90544	21.970598	23.088453	18.174451	19.435623	30.423624	18.472273	22.946724	81.572298
gene7655	RGS4	0.912647976797942	blue	4.918759	1.56411	1.019851	0.812013	2.089175	2.075726	1.477015	1.67844	1.68668
gene10787	LOC102165015	0.781841284718405	brown	3.588155	12.300022	4.031659	0.660739	7.693496	5.040676	6.184734	0.667571	3.177826
gene13424	RPS10	0.991922803361676	turquoise	269.480381	175.850675	182.756649	191.561599	183.216633	243.483439	204.213985	203.423595	654.699311
gene25745	TRIB3	0.829136426018042	blue	5.682406	0.671246	2.122481	2.416685	1.246059	2.226948	1.151989	4.954422	0.834708
gene16581	FXYD2	0.955324205001823	turquoise	2.901942	3.017284	2.661674	3.867263	1.213024	2.201157	0	1.489425	13.459814
gene9519	KLRK1	0.932841303635803	turquoise	2.62788	3.482553	4.601399	3.124494	2.008786	5.080224	4.84808	3.8157	12.232302
gene15773	LOC100524999	0.91032286054108	turquoise	2.100851	4.527984	2.514775	2.688443	1.960819	6.247969	1.599149	1.396539	10.771176
Sus_scrofa_newGene_12990	--	0.936255739223931	turquoise	9.433069	6.82698	5.210264	5.090266	4.161822	9.664045	2.161947	5.476251	18.039896
gene7298	CCNE2	0.825637314250319	black	0.5748	2.250648	1.326509	2.036914	1.319863	0.721515	0.572952	0.777622	1.40008
gene19230	AOC2	0.776965707841365	turquoise	1.788757	1.45954	0.88144	1.257912	1.900138	1.03296	0.670418	2.147992	3.34987
gene19950	VMO1	0.884215869326808	turquoise	6.303315	7.395614	2.594322	2.898091	2.767361	6.045405	5.599097	4.298697	12.821647
gene28129	LOC100624590	0.985242341313041	black	0	30.899459	0	34.842163	0	0	0	0	0
gene12577	LOC110261409	0.920296958228916	green	1.807821	7.197192	4.174828	13.326268	11.141599	8.500815	21.172243	7.353593	4.995499
gene23513	TECTB	0.933632050644988	green	0.386419	18.861389	9.185833	21.844063	18.845839	4.21315	35.738056	12.721027	8.537054
gene11851	NR0B2	0.753932443898541	blue	39.328266	4.323077	7.165297	8.619455	11.589329	5.553711	14.758368	28.669397	27.153639
gene24325	FAM171B	0.900945109016238	black	0.589568	1.945172	0.88291	1.990283	0.672353	0.69249	0.76468	0.816877	1.421601
gene586	DDO	0.729707509173106	brown	2.075863	8.440216	2.784382	2.224231	6.550059	3.382977	6.036497	2.879217	6.277782
Sus_scrofa_newGene_15938	--	0.748093272629937	brown	0.363224	4.126697	1.695275	3.258013	0.539817	0.8817	3.711953	1.887485	1.182337
gene7366	E2F5	0.786483311227307	blue	2.721214	1.25997	0.492808	1.794306	0.851779	1.209037	0.843706	2.296563	0.513486
gene15199	STAP1	0.958289262170765	turquoise	4.594725	3.173307	2.465479	3.106788	2.387958	3.362468	1.833012	2.598125	8.19651
gene10605	HAMP	0.93342871819627	turquoise	2.622246	6.12427	11.771171	11.389248	4.819633	0.793343	19.976377	4.838188	99.535233
gene25854	ROMO1	0.942023594883079	turquoise	16.936642	8.714719	12.12015	6.134299	11.89061	14.372113	12.486687	16.993586	41.824543
gene10652	COX7A1	0.988756008762415	turquoise	18.745213	11.063351	10.89361	10.302803	12.04659	16.458714	10.814089	9.854102	50.50391

gene81	ACAT2	0.847635948624139	brown	5.299779	33.650059	14.727136	19.123522	11.381536	10.527156	29.624334	6.815968	15.512717
gene1408	RPS29	0.936622472856982	turquoise	115.14289	21.490033	26.603984	18.454057	55.964569	64.570329	24.402789	54.968959	242.829638
gene24125	DAPL1	0.816835108348115	turquoise	0.716144	0.18976	0.791698	0	0.989637	1.138037	0	2.429705	4.066833
gene6699	FNDC4	0.932641970361228	blue	3.65168	1.341699	1.042458	0.656299	1.008474	1.62567	0.565643	1.490185	1.044299
Sus_scrofa_newGene_6198	--	0.895203818958434	black	1.045425	2.510943	1.281192	6.853184	0.867257	0.855013	0.526993	1.709303	0.618369
gene13804	LOC106504436	0.730229006467861	turquoise	0.836925	0.648776	0.318231	0.950452	0.629699	0.97235	2.128141	0.493803	2.58562
gene6354	SNRPG	0.946161413678511	turquoise	9.177893	4.136773	5.630471	5.20103	5.378457	7.449545	3.730198	6.71266	16.37328
gene8456	F3	0.724291956914159	blue	9.273233	2.401702	2.833302	4.171585	3.573694	5.018534	4.275758	10.514715	3.430074
gene10742	RPS16	0.991048417136597	turquoise	276.946014	172.57019	170.92363	195.606628	175.744019	236.80191	203.885605	195.517502	683.526062
gene10696	C6H19orf33	0.858188829672342	turquoise	2.716303	0.770738	1.307982	2.234414	2.048139	1.164386	1.4401	2.13711	4.968036
gene18785	FASN	0.961536531525461	brown	3.117734	51.671314	10.645468	9.550322	19.18848	4.356788	16.516336	2.560326	4.984661
gene11005	DBP	0.893998348739545	brown	1.031939	6.440816	3.374123	0.814894	1.740297	1.263512	3.180185	0.9578	3.178408
gene6987	GPIHBP1	0.789913080021647	turquoise	1.30843	1.057939	0.552414	1.973154	0.332388	1.118624	0.412326	1.267883	2.640302
gene22706	MMP11	0.878799079298234	brown	0.754902	5.357302	3.322036	1.213958	2.357493	1.024913	1.454389	1.307798	1.267511
gene14077	DHRS4	0.936579360097107	blue	187.126587	33.742992	62.213531	86.381546	49.081245	96.421692	78.059471	121.69751	79.320465
gene1857	TPM2	0.909248092488905	blue	58.998393	10.71408	9.054307	17.498763	10.189563	27.5208	13.938604	12.998989	28.664075
gene25458	MTMR7	0.942775144236912	blue	9.911668	1.195444	1.70804	2.454537	1.177246	1.358439	0.075855	0.828346	1.281967
Sus_scrofa_newGene_3118	--	0.846826083634352	turquoise	0.936063	1.799545	0.147689	0.467916	0.402042	1.207715	1.262292	0.497124	3.088765
Sus_scrofa_newGene_15259	--	0.850631007817584	red	4.254803	0.648801	7.793308	7.509931	22.601559	39.207658	25.600742	113.003668	39.611643
gene19562	RNF43	0.793520901857244	black	1.718974	3.164166	0.885608	2.911543	0.328558	1.160564	1.819929	1.695826	0.779061
gene13835	ANPEP	0.893631989553072	green	9.531644	50.774435	43.514876	37.259865	41.784521	17.625628	87.796251	22.250239	25.898725
Sus_scrofa_newGene_9897	--	0.955044238778415	brown	0.624927	3.74332	1.774376	0.725907	1.167741	1.117561	1.428367	1.068663	1.307626
gene6480	RPS27A	0.990803500569423	turquoise	353.074799	233.507187	240.492004	260.390167	225.472366	312.595825	211.270905	295.457489	863.513123
Sus_scrofa_newGene_8926	--	0.810999713622168	green	0	0.529587	1.584426	1.501199	0.73434	0.988293	3.262456	0.545392	2.05386
gene25885	NNAT	0.773921941994055	red	4.430204	0.674525	0.672435	2.191982	0.699444	7.841363	1.666939	7.291985	2.819604
gene25966	SLPI	0.955512727234369	turquoise	1.925727	1.373314	0.885329	0.741396	1.52774	1.493862	1.866521	0.774691	6.154886
gene6691	MRPL33	0.925326389316219	turquoise	9.787196	6.033074	6.712894	6.925889	6.520166	5.873069	4.589556	8.971345	18.636044
Sus_scrofa_newGene_5344	--	0.842066855787308	turquoise	0.393804	1.601196	0.998667	1.918187	0.826701	1.940214	2.1335	2.122541	4.623357
gene3678	HSH2D	0.771578624045898	turquoise	0.806273	1.998463	0.962821	0.628412	0.525699	1.405146	1.0768	0.742064	2.576267
gene15308	CXCL9	0.785099116799125	turquoise	8.11993	9.585075	6.838841	16.4007	4.558492	12.443645	4.919364	9.97889	20.649055
gene17390	LAMC1	0.900444952579239	blue	12.487956	3.9119	4.404159	7.156325	3.869979	5.152809	5.570775	3.577615	4.037809
gene22225	FDFT1	0.934413373645794	green	17.944557	73.090297	23.178127	63.77896	52.657117	37.340039	131.951147	39.040814	66.722545
Sus_scrofa_newGene_11314	--	0.831204492346717	brown	0.204938	2.921955	0.424285	1.499234	1.468598	1.165124	2.045256	1.460041	0.920243
Sus_scrofa_newGene_2802	--	0.716615200868886	turquoise	1.362138	1.362553	0.182867	0.880047	0.389544	1.201555	1.092445	0.730255	1.866462
gene17293	SUCO	0.983609832986038	blue	44.235077	8.182549	11.456154	16.577978	7.790538	18.538734	13.091071	21.225483	10.062752
gene8334	GSTM3	0.877565834335345	turquoise	3.194951	2.706452	4.150441	4.232264	2.491968	4.325796	5.745946	2.583114	9.920053
gene16187	P2RY2	0.96983244449643	blue	4.292325	1.007014	1.191732	1.942991	1.749026	1.573419	1.399076	1.955268	1.212719
gene11567	PER3	0.883957547762096	brown	1.247557	6.660414	4.49536	1.627376	2.209903	1.263547	2.49803	1.178358	2.197441
gene26292	WDR86	0.985445772704896	brown	0.86417	5.603254	2.156376	1.854708	2.031217	1.418897	2.36921	1.337196	2.29927
gene24539	IGFBP5	0.738960680518405	black	0.859165	1.055149	0.783551	4.021288	1.536935	1.839772	0.831463	1.191229	1.221308
gene339	HDDC2	0.874532387323672	turquoise	2.582607	1.183635	1.701173	2.127703	1.438247	1.025051	1.644561	1.761237	4.448951
gene4309	CFD	0.977269830607547	turquoise	18.78886	23.305824	20.160484	25.078049	17.873652	24.933695	20.859375	17.733219	67.776192
gene21531	COX17	0.809028986694209	turquoise	28.628555	9.082944	7.330251	7.047132	14.124117	17.269704	14.069339	17.267057	36.590565
gene18995	CD300C	0.928224496019405	turquoise	2.820779	2.523288	1.433262	2.738714	3.376939	1.461758	2.283929	1.50191	9.920212
gene26510	MEST	0.827246439678122	red	1.612142	0.550609	0.358594	2.018498	0.471926	2.214162	0.531467	2.377196	0.498871
gene28132	LOC100625207	0.971984009182626	black	0	6.447574	0	6.322617	0	0	0	0.015327	0
gene7287	UQCRB	0.837520570483904	turquoise	72.579874	30.105736	32.638992	31.962972	34.556828	39.339612	18.94458	39.074321	91.343735
gene14516	FOS	0.893628839705684	brown	30.991777	151.548126	5.964465	2.996882	8.346496	19.316259	15.166135	4.861709	4.265257
gene13723	BCL2A1	0.905653834327704	turquoise	3.425748	2.160486	1.473295	1.719793	2.394827	4.251864	0.945358	2.315547	6.596659
gene16503	COLCA2	0.781513727490889	brown	4.843766	18.724943	3.427425	9.366433	9.369285	7.588655	8.566579	13.060793	6.948276

gene24992	HMGCS1	0.939105216170067	green	10.711837	77.240281	17.624735	62.35567	30.320671	22.645713	140.951444	20.005351	47.328346
gene26411	NDUFB2	0.916083891120239	turquoise	33.121746	10.385635	10.698682	11.972304	13.68098	21.399931	10.601249	14.540517	51.685425
Sus_scrofa_newGene_15118	--	0.816343442733716	turquoise	12.266132	4.905126	4.156915	6.585947	5.124393	8.714386	3.571275	6.776211	14.493
gene7997	S100A6	0.84301271384104	turquoise	2.740325	0	0	0	0.685641	0	1.054017	3.928208	1.213766	7.363243
gene23295	CYP2C34	0.985121493307121	black	9.738923	269.813416	51.515392	476.766724	24.62722	21.669285	42.116859	9.660285	22.038925
gene16253	LOC102162715	0.867747520872707	black	0.383721	1.644035	1.448546	2.434708	1.011213	1.072274	0.964431	0.520857	1.26803
gene6144	MAP4K4	0.969994780547856	blue	22.780739	6.432693	6.689331	7.972491	9.56444	8.005334	6.621422	9.942091	6.369263
gene20655	ITIH4	0.795958293386123	red	1013.86214	955.486561	893.564534	1617.646002	1366.83949	1420.605595	2923.374362	3691.348556	1228.241976
Sus_scrofa_newGene_15842	--	0.858618753972828	black	1.117859	1.849358	0.262607	2.189219	0.252667	1.102699	1.069205	0.701143	0.940605
gene7274	RPL30	0.991573953603975	turquoise	211.99893	137.189368	132.316	170.484766	153.994838	195.106588	139.033995	177.918535	554.884667
gene1330	CALML4	0.768536436580857	turquoise	1.980353	2.698007	4.401356	5.833784	2.394819	3.177718	2.082267	2.243719	8.044572
gene19636	CCL3L1	0.902880542998772	turquoise	0.93056	2.527051	2.182783	1.54336	2.858635	3.773386	1.560177	2.499633	7.759502
gene25490	C17H8orf4	0.955156142430811	blue	25.14468	6.772228	5.952775	4.217506	4.243472	6.7713	5.729402	8.735683	9.148364
gene15514	ANXA5	0.986165977096263	blue	58.156345	11.149582	13.942738	15.068054	13.030424	17.733784	11.577105	25.737461	19.195435
gene2679	SLC25A45	0.880193603655335	blue	9.899606	0.589811	1.900295	4.298865	2.528166	3.874562	2.271969	7.651685	1.715692
gene8790	MFNG	0.912984929409313	turquoise	2.316351	2.32652	2.029019	1.826111	1.037004	1.965828	2.347553	1.203166	4.900267
gene16590	JAML	0.938219060476956	turquoise	2.951575	2.500778	2.163012	1.621607	1.256135	3.431484	2.750815	2.682964	6.263042
gene30300	S100B	0.89386689485712	turquoise	2.42767	3.190163	1.945483	1.600952	1.107782	2.137425	1.682755	1.996853	5.14611
Sus_scrofa_newGene_879	--	0.836115183273435	green	0.375748	2.661196	1.980104	1.616095	2.447431	1.976127	4.921488	2.022237	3.131125
gene21078	PFN2	0.856761890494089	red	1.843229	0.515115	0.585274	1.074056	1.089907	0.953877	0.772187	2.881732	0.67301
gene17851	CCL19	0.896871695751589	turquoise	6.428299	14.428729	10.909482	8.409687	17.889172	25.884951	13.167758	10.606402	47.013351
gene27834	MBNL3	0.804799797830925	brown	10.38063	69.33258	27.719442	52.862508	22.927629	29.515347	41.265123	37.153026	15.359613
gene23302	CYP2C49	0.980798801739764	black	129.2686	582.164917	200.88179	805.8396	103.35228	98.768806	172.9328	127.841179	95.188889
gene19382	RPL23	0.98828305725733	turquoise	438.796234	255.388107	244.015564	268.308258	279.851959	384.764801	214.014236	323.314545	1090.363403
gene28419	DNLZ	0.866387090255491	turquoise	1.176082	0.257392	0.968601	0.386623	0.858783	2.234836	0.701617	0.29934	3.225002
gene5503	LOC100522011	0.91592458187113	turquoise	1.224509	1.128134	1.452254	0.285418	2.328689	1.836143	1.373232	1.694617	6.432439
gene8969	PFDN5	0.982001759126863	turquoise	23.220423	17.341784	18.929373	12.402666	15.40767	23.396034	16.165716	16.911528	56.831341
gene27752	NDUFA1	0.930072524922163	turquoise	11.743997	4.821872	6.724684	6.11978	5.853786	8.929079	5.062444	7.538609	18.632698
gene10826	RPS19	0.9931935386525	turquoise	269.847495	172.292492	169.126784	204.825441	190.210562	251.198869	177.608311	198.638037	616.743579
gene3257	SAA3	0.899916262481364	red	45.904069	30.875497	24.779097	106.107064	77.995299	87.168844	116.997436	251.901552	117.51785
gene25709	TGM3	0.908979394877714	blue	7.891141	1.04072	0.20713	1.567526	0.329537	0.426507	3.161861	1.801735	1.271486
gene4713	LOX	0.712323955599016	blue	4.15899	1.146706	0.833411	4.065266	1.885661	2.401308	1.784002	3.016753	1.924563
gene4663	NREP	0.843876112071362	brown	1.615288	9.418594	6.966642	2.892251	3.569395	3.527223	6.48565	1.699456	4.234964
gene27417	GDPD2	0.844371844996438	green	0.827338	0.576718	0.241046	2.030496	0.898109	0.665153	3.026307	1.760153	0.621145
gene11135	NDUFA3	0.921831439841788	turquoise	7.253716	1.699027	4.09989	4.831337	4.525492	6.203731	3.98737	4.986575	12.994368
gene4090	PET100	0.969182132797461	turquoise	5.576601	2.963172	2.45527	1.800863	3.595323	3.996832	2.119974	4.160064	15.308225
gene17713	ATP6V1G3	0.848947673513507	turquoise	0.699623	1.164889	0.302477	1.375395	1.808427	2.38172	1.24195	0.595355	3.915715
gene20067	RPL26	0.986063797599369	turquoise	342.375538	209.368713	197.365832	243.850447	224.939701	307.836761	152.974219	270.102553	835.77879
gene16894	KLHDC8A	0.755363759613761	green	0.22342	1.997995	1.158353	0.388533	0.798453	0.651961	2.632465	1.114107	1.092431
gene14616	NDUFB1	0.916702233582762	turquoise	4.342559	1.652243	1.441143	0.68755	1.635164	2.679673	0.886536	4.179716	9.86712
gene24058	PPP1R3B	0.848898047174765	green	3.222703	8.177735	1.787443	9.079608	4.809548	1.698607	13.824393	8.489235	1.644329
Sus_scrofa_newGene_13826	--	0.955622234661767	green	0.180536	1.37777	0.815048	1.733534	1.0273	0.610298	2.626922	1.463673	0.958194
Sus_scrofa_newGene_9044	--	0.72168145260848	blue	150.247559	55.725571	121.904167	67.654083	60.436169	64.503036	27.846626	70.647003	54.874363
gene20926	CHST13	0.866649664837025	blue	11.294749	2.222459	2.806051	5.103185	4.261899	7.498409	4.790198	8.764307	3.71969
Sus_scrofa_newGene_5163	--	0.873182333930232	black	0.37688	2.120002	1.550597	3.439652	0.95689	1.475758	1.905387	0.933249	1.25165
gene25942	FITM2	0.96977862019618	blue	12.818777	3.696992	2.874453	5.002525	2.830931	4.568654	4.445441	4.011353	3.013108
gene27248	TIMP1	0.72650094788207	blue	6.208397	1.990837	1.599707	3.045589	1.198296	3.166118	0.618714	2.547647	5.270478
gene11766	C1QA	0.951239563241435	turquoise	260.471851	272.475992	287.049385	367.073051	217.496204	375.066463	243.669358	184.126625	738.320862
gene6596	MORN2	0.937383619940044	turquoise	5.33414	6.603648	4.197698	6.969595	4.799384	7.808762	2.94352	7.432869	16.03297
Sus_scrofa_newGene_12242	--	0.939810730940039	turquoise	1.580998	1.202511	1.622584	2.012289	1.557765	1.566378	0.77907	2.023496	5.100084

gene4035	CNN1	0.772485541127568	blue	8.291378	1.784319	1.296699	6.51475	1.522115	5.767737	2.024873	2.421467	2.834549
gene23293	CYP2C36	0.801592210747903	black	8.100445	31.065519	32.512856	45.222702	12.971349	12.913195	24.241621	8.913729	11.093724
gene5779	PPL	0.799467713348037	red	4.4284	2.318887	1.146735	4.497984	1.842209	5.705213	3.373347	5.654383	1.370919
gene16247	WNT11	0.818968581492935	brown	0.857985	3.239433	0.422065	0.831122	0.757293	1.846366	1.236333	1.032915	0.803355
gene10640	TYROBP	0.985150102159098	turquoise	23.905246	13.907588	16.236138	17.439018	12.593957	30.545196	20.914171	13.961661	70.010599
gene28390	PTGDS	0.864872544024176	green	6.461081	8.932969	2.368598	9.368479	9.062883	9.471851	21.767519	11.148808	9.7968
gene13397	RPS18	0.978090737006196	turquoise	299.534393	160.377029	138.976456	177.535995	169.198914	244.456619	165.02124	194.234772	590.871094
gene16868	SNRPE	0.965970425991765	turquoise	18.435772	7.902579	11.436056	8.599726	13.745393	17.012316	11.392755	14.964985	45.219658
gene16565	ZPR1	0.952523042793638	blue	83.875732	18.385658	36.981792	31.738764	25.152508	38.246258	19.492826	31.350002	28.31282
gene1701	PTPRD	0.903405995067496	brown	0.789564	9.592438	5.759375	5.03066	2.914308	1.902707	3.698303	2.574369	2.394283
gene13292	CLIC1	0.985227412739882	turquoise	9.433577	8.198709	9.42214	7.453906	6.516156	10.762436	7.335091	7.656541	23.060995
gene13243	IER3	0.942980539798889	blue	10.865586	2.766906	0.553487	0.976822	1.89829	1.526079	1.456449	2.426264	2.032489
gene7741	TSTD1	0.771549930957045	turquoise	0.93326	0.66669	1.31926	2.590427	1.584356	1.061622	0.520253	0.219509	3.839649
gene15901	GVIN1	0.807643644215118	black	0.65297	3.212032	1.08979	2.948607	0.54855	2.777172	0.731935	1.235903	1.179168
gene25828	TP53INP2	0.793121578450849	blue	17.1261	2.329459	10.608022	2.35552	3.994728	2.939731	2.606497	4.004647	4.646838
gene6415	MDH1	0.988258269136436	blue	136.964233	36.356331	37.779934	43.666214	38.199684	46.852089	45.99015	63.173805	51.258759
gene8961	SOAT2	0.798503982735101	green	1.990879	12.624095	4.780776	15.985192	10.250298	6.597034	15.84452	4.926601	7.091182
gene6154	RPL31	0.99008472785515	turquoise	227.034958	148.863708	142.172073	159.773773	157.142578	214.81395	130.741669	203.222534	626.599304
gene15766	MRPS18C	0.896475987289199	turquoise	2.690073	1.407208	1.72835	1.649229	1.894432	2.396423	1.259306	3.534471	5.981928
gene20417	HIGD1A	0.941226560942841	blue	152.951462	26.731378	50.155754	62.741605	43.557693	77.850448	25.159598	55.53035	62.278987
gene12601	LOC100524873	0.825542959877271	turquoise	77.713037	24.526875	31.902578	30.290335	26.46109	39.442948	23.117188	37.133774	93.359129
gene681	COX7A2	0.899647946727389	turquoise	48.762363	21.090031	23.367598	22.412647	23.870939	35.375225	14.615822	29.305346	71.283531
gene9180	ARHGAP9	0.766889069451885	turquoise	0.708551	1.628497	2.164266	1.764336	1.154738	1.949391	2.34418	1.02362	3.831262
Sus_scrofa_newGene_1264	--	0.863416417529091	blue	5.72025	0.23608	2.041748	2.313074	1.709512	1.603595	0.721354	1.176997	2.669449
gene2553	CPT1A	0.918581669788157	blue	181.216034	40.678593	91.838524	45.226353	49.451958	59.855995	49.441879	78.868843	45.866482
gene12377	CTH	0.836692597387767	blue	624.773804	126.403358	330.640778	210.67514	242.556046	307.680908	103.870346	167.730316	288.152344
gene4544	COX7C	0.983434416365634	turquoise	96.012212	44.443308	54.905222	58.909182	45.880707	76.289524	34.120293	69.109347	250.282962
gene20882	LSM3	0.87962920212006	turquoise	28.430905	14.348791	10.023739	13.303951	13.587963	24.92844	9.845291	11.708591	39.850537
gene11016	LOC110261360	0.926963689932369	red	2.436856	2.250745	0.860011	3.780574	1.506959	2.941065	4.132538	7.634101	1.274472
gene13763	RPS17	0.988231401208713	turquoise	217.077606	125.634659	121.091354	140.531921	154.394928	191.442581	128.503845	166.312408	564.186951
Sus_scrofa_newGene_3609	--	0.80705378132876	green	0.217114	3.820296	0.705303	1.078721	1.373123	0.925549	5.584652	3.28104	1.358677
Sus_scrofa_newGene_2498	--	0.860735835685464	brown	0.741354	3.843721	0.638622	1.588712	1.237039	0.793019	3.037462	1.684217	0.999554
Sus_scrofa_newGene_3488	--	0.744927959941346	turquoise	0.584853	0.57556	0.692937	1.593756	0.579801	1.250961	1.396263	1.299496	2.307442
gene27110	GK	0.9550832181601	blue	54.833792	19.878207	18.293175	24.119803	11.491825	27.119367	17.503047	20.466791	14.606312
gene14968	KLB	0.953429352732178	green	1.969631	16.713026	10.062662	13.989406	8.302539	3.770555	30.447506	6.695124	8.984072
gene19814	RILP	0.749274046562756	red	0.503465	0.167614	0	0	0.017889	4.217614	0	4.071749	0.214556
gene14050	CIDEB	0.885417570496597	blue	322.281227	71.967478	108.394016	123.816788	112.16328	236.931805	94.815375	160.883537	161.066189
gene16281	THRSP	0.720445685492385	brown	0.129094	14.032535	1.440142	8.435197	3.540672	2.125752	16.119488	1.854524	0.882417
Sus_scrofa_newGene_2751	--	0.893722609909922	turquoise	0.21632	192.21336	38.499592	45.133967	10.566659	178.8243	179.034823	0.133396	568.049404
gene23079	PLAC9	0.964915665767431	blue	5.655984	1.840207	1.767101	2.924531	1.742759	2.700786	1.30315	2.646545	2.015346
gene22616	UQCR10	0.904368164901717	turquoise	41.635506	18.897608	19.872026	15.980386	26.212788	33.383251	19.119585	25.363247	63.554024
Sus_scrofa_newGene_14857	--	0.942165717827215	turquoise	8.109991	11.580159	5.640316	12.456015	8.345555	10.20263	9.39145	11.572626	28.963385
Sus_scrofa_newGene_3801	--	0.88491097538588	black	0.899605	6.383865	1.561917	5.674833	1.479599	2.604376	3.537704	2.506835	1.9936
gene7843	CD1D	0.974726943451486	turquoise	3.85506	5.450306	4.633428	5.117837	4.003383	5.928392	4.924071	3.800888	17.310337
gene13400	PFDN6	0.965422795580286	turquoise	14.711075	8.129735	7.242902	10.163387	8.018616	14.989159	7.492923	11.735479	28.464844
gene2876	LOC100524972	0.962742929681228	turquoise	25.682661	27.184442	27.338068	19.071363	16.748583	31.457061	17.60214	23.110974	63.22484
gene9541	KLRB1	0.821582103025445	turquoise	0.660838	2.340737	1.898172	2.629971	0.240625	1.187878	2.980489	0.588153	6.546133
Sus_scrofa_newGene_7527	--	0.759912412749754	blue	6.512032	1.078932	2.168061	1.267158	2.910339	1.244727	4.31102	4.042348	1.525576
gene19977	ACADVL	0.851218655751867	blue	96.087919	19.547654	31.613486	42.569486	37.885875	61.889517	32.99668	81.878376	37.927783
gene9750	SLC38A1	0.963375464823177	blue	10.604239	0.818889	2.48514	2.03554	2.270035	1.531528	0.830986	2.844345	1.717212

gene1086	THBS1	0.940144959336309	blue	12.838902	5.104253	2.29689	5.463982	2.7952	4.810921	3.620388	3.923147	3.868748
gene6099	DUSP2	0.902336862355767	turquoise	1.538412	1.209161	0.52725	0.895946	0.478183	0.767673	0.91768	0.662645	2.645586
Sus_scrofa_newGene_1656	--	0.941842918828662	green	0.345587	0.679538	0.347549	1.442048	0.810466	0.392798	2.801544	0.983168	1.317807
Sus_scrofa_newGene_8320	--	0.700328852423242	black	1.944036	3.205077	2.148918	2.656961	1.713216	1.272236	0.651399	0.268183	0.445991
gene2872	MS4A4A	0.934312547484796	turquoise	11.174021	20.162951	13.456795	18.344437	9.426607	20.19074	12.051131	11.122835	42.313083
gene28846	CKB	0.863941536638163	blue	6.411486	1.82409	1.44563	2.314793	3.000828	2.921683	3.07656	1.625019	2.923665
Sus_scrofa_newGene_4274	--	0.774973891103541	green	0.693096	2.327798	1.635754	1.371872	1.405812	0.979963	3.140339	0.76602	2.184539
gene16920	IL10	0.899181912135546	turquoise	1.028499	0.796066	0.860195	1.115888	0.679497	1.746082	0.206863	0.689816	2.63501
Sus_scrofa_newGene_16538	--	0.97285590820317	turquoise	0.753285	1.396962	0.401066	1.350062	0.536861	1.529511	0.891601	0.40204	5.475099
gene7059	KCNQ3	0.830032794784203	brown	0.27407	2.340729	0.767041	0.739782	1.608027	0.518151	1.906081	0.756305	1.02405
gene10297	MT1A	0.831181649196428	turquoise	373.86322	659.390747	611.167786	450.023926	186.950699	476.645935	394.077026	217.690063	1136.860596
Sus_scrofa_newGene_13369	--	0.778115450933689	brown	0.157315	3.051947	1.602186	0.603708	1.563978	1.382076	0.688529	1.671357	0.917142
Sus_scrofa_newGene_9054	--	0.858096763751444	turquoise	1.260524	1.137461	0.805126	1.245349	0.912371	1.124812	0.132252	1.322808	2.564243
gene13681	GSTA4	0.826841099487151	black	10.322721	44.030378	19.867655	41.775239	34.129093	17.805934	11.482565	15.219289	15.909594
Sus_scrofa_newGene_7145	--	0.926899947305464	brown	0.681384	3.689619	0.898025	1.891997	1.587764	0.995569	1.688997	1.655309	1.566768
gene6299	WBP1	0.942587343248203	turquoise	6.149861	9.927062	7.178047	11.143111	8.43232	12.50541	7.924604	8.549489	24.110533
gene4018	SPC24	0.801330846922301	turquoise	1.408064	1.18606	0.810852	1.705067	0.423747	0.936739	0.52209	0.764449	2.356535
gene20283	NR1D2	0.719902522666007	brown	4.876672	23.565845	23.364342	6.597709	9.00106	4.344871	9.361023	7.233165	9.582176
gene23069	RPS24	0.989560753136096	turquoise	402.883082	267.766713	232.109713	273.359455	273.958651	377.120321	190.393637	288.271064	980.551791
gene786	ONECUT2	0.845913220655946	brown	1.906332	5.706676	1.825827	3.653795	1.883657	2.213686	4.145037	2.159107	1.254883
Sus_scrofa_newGene_15055	--	0.793467984477059	brown	0.200962	2.603741	0.344482	1.653559	0.854219	0.659821	1.965255	1.169127	0.181673
gene1548	ISG20	0.807135408084518	turquoise	3.205448	2.80031	1.331413	1.928535	1.074304	3.806788	1.6175	1.521432	4.672134
gene13414	C7H6orf125	0.931691879763745	turquoise	23.870197	9.00205	12.993166	15.211018	9.265434	20.350206	14.467577	14.949903	39.01791
gene7995	S100A4	0.935161604870661	turquoise	1.049188	1.033328	0.978648	0	1.28807	1.083007	0.693054	1.031753	4.658069
gene27266	RBM3	0.765091823471172	turquoise	26.647453	10.012958	8.746021	11.180342	9.366996	16.270674	8.410683	12.739152	27.786959
gene16550	NNMT	0.732075917215234	blue	487.435883	101.284966	329.928436	146.451553	241.416611	185.055557	179.001556	420.538788	179.559021
gene18442	SLC25A30	0.835294764174661	blue	135.90254	8.669774	26.406638	49.598196	21.135845	90.079073	51.855589	108.876113	45.705096
gene8874	TUBA1A	0.926028007094805	blue	35.389775	9.136658	11.959378	12.922892	9.423661	14.452512	13.561474	14.302269	20.275734
gene3323	ADM	0.7021479020521	blue	8.338251	1.637425	3.836867	2.709277	2.943745	3.79986	1.497915	1.889061	6.725068
gene8848	CKAP4	0.704278494675661	blue	6.967076	3.356819	1.940829	6.202542	2.155896	2.633873	3.548232	2.774472	1.435747
gene11017	FGF21	0.864601731191272	red	6.492475	0.498496	1.019215	1.984792	1.619776	4.187788	12.10995	27.506655	2.902334
gene19644	CCL5	0.924860279882493	turquoise	18.836517	13.305677	16.393892	8.890631	11.825792	17.248724	20.219498	11.619968	41.660057
gene19634	CCL4	0.966599001620345	turquoise	5.086092	6.433402	4.686236	5.057284	4.134253	8.584893	5.612359	5.200281	15.32909
gene8873	LOC100127131	0.980855676618849	blue	44.82468	5.829467	10.117929	15.893579	9.422029	17.191288	15.252092	21.981941	13.586727
gene25335	ATOX1	0.968641928581395	turquoise	29.002237	25.77268	29.779184	25.816666	30.923334	33.278328	27.984179	34.757294	82.414978
gene1660	PLIN2	0.962913221295687	blue	383.364761	7.786508	10.790402	78.028593	13.9968	92.183463	34.045714	203.940725	42.490829
gene15622	NPNT	0.893331486289269	brown	1.360984	9.730793	2.353595	3.591579	5.62939	3.157098	2.979039	2.196805	3.077468
gene17875	AQP3	0.712246936839047	blue	4.315179	1.003027	0.445916	1.153163	1.461709	2.965671	1.529383	0.869706	3.223508
gene24536	RPL37A	0.984729618683541	turquoise	69.107725	26.706326	29.938884	34.424263	37.00863	59.625009	30.470787	46.488425	182.34542
Sus_scrofa_newGene_14686	--	0.748966783471993	green	12.885037	13.946679	11.648937	50.718915	18.537921	19.126614	44.602225	14.732147	26.285402
gene7259	COX6C	0.983175467541212	turquoise	64.758334	32.018894	37.966789	31.06838	41.914835	58.553795	26.994606	41.360241	173.082395
gene14964	TMEM156	0.909041307335866	turquoise	1.295129	2.025988	1.524053	1.846109	1.586944	2.972382	0.703369	1.745975	4.829184
gene11758	LOC100627283	0.978143922720527	turquoise	1.557611	1.207662	1.298022	1.167495	1.479445	1.834956	1.682742	1.248845	4.93105
gene28896	LOC396781	0.773753235273735	black	4.606705	8.738441	4.254741	19.493448	4.019512	6.285408	9.538507	7.099668	11.348137
Sus_scrofa_newGene_12229	--	0.899690305548118	turquoise	1.030848	1.3281	0.30214	1.523534	0.762619	1.79279	0.930298	0.924499	3.021035
gene3609	GDF15	0.916731623139429	blue	5.260872	0.900262	0.459559	0.592211	0.208574	0.544573	0.040068	3.045285	1.056215
gene10990	EMP3	0.91948291781459	turquoise	4.438897	1.364808	1.119596	1.974654	1.859568	2.807157	2.339446	2.836057	7.501382
Sus_scrofa_newGene_1759	--	0.865365409790375	blue	4.797188	2.149529	1.640566	0.909205	0.622635	1.839025	0.552605	1.131015	0.70354
gene25038	GZMK	0.951290757580744	turquoise	1.395264	1.481572	1.703372	0.870833	0.926073	1.609342	0.750115	1.47996	4.400908
gene19222	RPL27	0.976352653942402	turquoise	172.47963	93.764702	90.04451	105.94281	109.193016	146.077805	90.765495	133.835312	355.27417

gene14653	LOC106504547	0.973672152163448	red	1602.81604	519.689331	712.17511	2950.70874	1174.168457	2515.437012	2077.426514	4409.958984	1267.260986
Sus_scrofa_newGene_14893	--	0.940057628770912	brown	0.053464	4.423948	1.345161	0.327366	0.829493	0.365363	0.933408	0.712162	1.690345
gene11545	RPL22	0.993964138763118	turquoise	139.575287	102.081963	91.378738	107.349243	104.76355	148.949463	93.14917	130.046707	425.58432
gene7421	LY96	0.988560091539314	turquoise	6.238776	4.863511	4.136394	4.268963	3.063777	6.274475	2.805559	5.851551	19.215032
gene4171	PLIN4	0.966921537000788	blue	5.499472	1.212401	0.317375	1.420271	1.350739	1.67438	1.111492	1.380748	0.80792
gene15603	RPL34	0.963444667736733	turquoise	177.685493	35.682622	95.540679	100.785507	130.206342	148.354917	80.873706	163.404707	552.198876
gene16618	RPS25	0.99519350162952	turquoise	141.587083	96.41862	85.006126	104.701706	94.643539	130.990544	84.821596	99.28373	345.468353
gene26773	CYCS	0.90992995907543	blue	94.635925	22.120852	34.33112	26.755451	21.848019	32.725704	12.551933	21.272942	40.020885
gene4682	CDO1	0.738331045113429	blue	210.030396	129.682968	93.066185	43.288208	58.334232	35.47673	69.187866	85.467468	23.515196
gene16527	IL18	0.93367939643733	turquoise	3.568862	2.765642	4.630207	5.41223	3.963363	4.830255	4.254134	5.984784	13.687031
gene12355	SLC44A5	0.798914457194872	brown	0.33895	4.371185	2.45884	0.758531	2.069321	1.04346	0.317126	0.419218	1.725772
gene2563	CHKA	0.845810245780418	blue	16.648405	7.879057	6.064387	4.651541	5.798685	3.940543	7.243985	8.072789	2.396282
gene3999	S1PR5	0.892297398912544	blue	3.71136	0.968776	0.662425	1.599538	1.756878	1.382459	1.809976	1.834057	0.699134
gene13891	LOC100156967	0.961775747212636	blue	103.772194	23.753864	29.00271	37.09827	39.705704	49.831844	40.614632	43.028294	47.335201
gene1479	LGALS3	0.895721336204895	turquoise	2.189304	0.939966	1.084562	2.044956	1.104539	1.487165	0.194447	2.007515	4.615766
gene13274	LST1	0.989348859749852	turquoise	0.782301	0.705204	0.569756	1.10735	0.635725	1.540988	0.658646	0.807807	4.363857
gene5364	SERPINE1	0.944817834869321	blue	6.781785	2.088657	1.423833	1.329352	1.23595	2.302802	1.193201	1.514178	2.089252
gene628	FAM26F	0.797152318726936	turquoise	1.472994	3.058811	2.18998	2.501788	1.687307	5.609361	2.900294	2.272881	6.508741
gene15774	PLAC8	0.88543580187613	turquoise	13.194812	23.859493	21.320309	21.612844	7.530462	34.612141	6.946982	11.655622	57.001354
gene27597	RPL36A	0.977995135059644	turquoise	52.40641	26.037868	24.479906	29.419327	32.331257	42.057098	10.839932	33.586063	142.719498
gene16592	MPZL2	0.967121676160765	blue	26.45871	7.981424	8.858404	9.899778	7.048797	11.9455	10.516376	14.7039	6.124546
gene21650	RPL24	0.978237263574098	turquoise	169.156616	97.933167	88.60981	110.706772	99.144508	136.385269	90.57975	114.660927	320.822662
gene10296	LOC102166944	0.857654484478986	turquoise	1.735237	169.544586	197.679865	132.111252	74.900726	240.328537	195.813084	4.4829	582.817978
Sus_scrofa_newGene_13012	--	0.827879667502718	brown	7.701146	24.324275	8.777525	10.159302	5.795254	5.13602	5.121514	13.262278	6.376642
gene25865	MYL9	0.902839281408151	blue	31.690334	5.916111	6.328876	6.859075	6.786582	12.763233	12.656901	6.575397	13.827939
gene6258	TMSB10	0.990175694135107	turquoise	235.758133	149.53212	163.679352	170.331085	169.422684	280.083923	177.395828	241.96936	800.415771
gene5976	MCRIP2	0.974293785590764	blue	77.606493	18.875095	18.569124	23.502942	26.048649	36.589713	22.939263	26.87466	25.321146
gene21068	TM4SF18	0.951581423178923	turquoise	4.530823	3.441433	4.011152	3.159788	2.172215	4.189502	4.129652	2.427724	9.730148
gene10535	PLEKHF1	0.9532047201717	blue	25.681468	2.858796	7.108771	8.707307	4.91608	11.195645	7.363951	15.663454	7.495822
Sus_scrofa_newGene_1509	--	0.962482758875571	blue	13.043884	0.008126	0.086111	0	0.067116	0.026846	0.009454	0.043956	0.04418
Sus_scrofa_newGene_11551	--	0.794920897498008	green	0.613668	1.552713	1.776665	1.35156	1.648334	0.462223	3.54409	0.838812	2.45424
gene9128	ZC3H10	0.982278903852884	turquoise	37.699613	16.791906	17.404565	16.818043	18.894505	27.477365	17.481244	25.882282	100.092106
Sus_scrofa_newGene_3392	--	0.965042441888749	green	0.455813	2.343342	1.703301	1.918637	1.617285	0.869988	4.783877	1.39847	1.331877
gene22019	PDXK	0.863605986107543	blue	28.570747	7.86669	8.848481	4.051561	10.148063	12.316015	6.403675	15.10698	16.560547
gene6556	SLC3A1	0.808039437013378	green	1.94182	1.413917	5.715695	4.825218	4.340707	6.081207	13.612474	5.64613	6.289425
Sus_scrofa_newGene_2858	--	0.742881839380641	green	0.401878	0.703496	0.999548	2.198851	0.42685	0.542787	1.973849	1.49811	0.916232
gene19955	CXCL16	0.97504338398325	turquoise	73.340788	60.279049	46.436314	51.469402	44.193071	66.697902	61.013535	40.76222	151.167576
Sus_scrofa_newGene_10863	--	0.769100263156773	turquoise	2.540598	1.154224	1.208218	0.975073	0.381111	5.237063	0.906949	6.274085	8.374723
gene21828	ATP5J	0.903755804934424	turquoise	78.039948	36.882522	28.752741	36.549599	36.237214	52.121275	30.717891	49.165821	112.518723
Sus_scrofa_newGene_14543	--	0.880005448250324	turquoise	0.926063	4.240371	1.87328	2.727371	0.802457	4.20902	2.289698	0.254017	9.223906
gene955	C1H15orf48	0.874490823350806	turquoise	0.954397	1.912223	0.445538	0.485039	0.747319	1.986469	0.447607	1.059849	3.51998
gene4531	RPS23	0.982846383959252	turquoise	222.651825	120.883476	140.12645	172.486557	150.228195	187.404816	152.106918	176.66156	495.315979
gene8003	S100A9	0.713782132691532	turquoise	2.895778	1.844879	0.143848	0.216366	1.020936	2.445837	2.042124	2.722366	4.177461
gene3254	SAA2	0.882541991689391	red	150.370377	282.42749	124.471313	678.665222	283.676941	854.749146	606.782898	1823.127197	970.873413
Sus_scrofa_newGene_3419	--	0.873981903223864	turquoise	0.784751	2.123058	1.633948	1.191467	0.952288	1.818283	0.938704	1.758331	4.032011
gene13676	TMEM14A	0.951396381873211	turquoise	1.892796	1.482897	1.73247	2.042185	2.215333	2.72053	1.643823	2.953833	6.542588
gene11052	RPS11	0.992576308573866	turquoise	353.744743	274.207025	276.577309	312.399341	257.102018	391.907036	345.078076	298.477522	964.751343
gene10060	CYBA	0.974767418940679	turquoise	12.740234	12.519911	13.925194	15.55407	12.426714	18.303372	14.85127	10.821822	39.514542
gene14848	LOC100525472	0.817092426243691	blue	6.908644	1.104974	0.123957	4.335617	0.876686	0.913031	0.368271	0.078013	0.224173
Sus_scrofa_newGene_4150	--	0.729118110179685	brown	0	2.260187	0.603694	1.075039	1.439664	0.576123	1.967353	1.644442	0.571798

gene148	AKAP12	0.960909115395252	blue	18.755817	3.762785	3.988822	4.17749	2.873337	3.594097	4.668358	3.879652	3.036486
gene26829	NPC1L1	0.702364552694697	blue	12.833828	1.800818	7.946676	2.23052	7.08121	3.750502	3.412711	1.98472	3.666174
Sus_scrofa_newGene_14036	--	0.881363347427813	blue	5.291596	0.161394	1.845413	2.065624	0.786845	1.375144	1.120187	3.790342	0.93214
gene23856	GYPC	0.891604269318781	turquoise	1.70798	0.67322	3.593432	1.810545	1.706148	2.379186	2.54436	1.603057	7.804404
gene12089	TUBB6	0.832454622476346	blue	4.105249	0.859399	1.270943	1.758495	1.059527	1.6795	1.041778	1.377571	2.909846
gene28886	LOC110258364	0.942301759579897	turquoise	1.969397	2.293119	1.729596	0.985787	2.260404	2.462388	3.392189	1.528951	9.871466
gene290	CTGF	0.76607325428819	blue	12.284409	6.506601	4.948885	3.573659	1.993572	4.381711	3.726032	1.81759	5.989416
Sus_scrofa_newGene_3061	--	0.749916412634386	turquoise	17.073324	6.27327	6.119834	4.766185	5.716576	8.673809	2.907965	5.340363	17.788419
gene14199	LOC106504487	0.906449807061053	turquoise	0.917006	1.698008	0.903116	1.469185	0.820102	1.468796	0.619847	1.154064	3.143735
gene17107	TOMM7	0.983325473467775	turquoise	14.219536	8.493408	5.493537	9.839569	9.340006	12.298858	7.052568	12.189405	39.05645
Sus_scrofa_newGene_11913	--	0.748528013141623	green	0.149692	3.021796	1.593047	2.790197	1.298295	1.632877	4.540595	4.154031	3.127898
gene20950	SLCO2A1	0.847036244558138	blue	15.94518	2.367919	6.066833	3.243102	3.721043	9.693079	3.298791	1.928295	3.635973
gene13225	SLA-3	0.875767743710849	turquoise	110.913201	67.454945	50.547229	54.85797	43.041225	102.028554	41.261988	118.764894	194.670237
gene9125	RPS26	0.988950301183621	turquoise	330.739532	199.204498	203.886002	224.678558	217.819687	316.141876	257.090424	287.016907	883.077148
gene18905	JMJD6	0.975022316330231	blue	2.917233	0.766011	0.646427	1.205668	0.583385	1.170972	0.789471	1.373699	1.224396
gene5798	VASN	0.757493717441413	blue	12.230536	3.613933	2.661435	8.972971	5.509153	4.362786	7.389662	9.04456	2.517657
gene12145	MYOM1	0.875171809331882	blue	6.293038	1.286673	2.092269	2.203724	3.156347	1.963439	3.334927	2.522061	1.916833
Sus_scrofa_newGene_5453	--	0.785606666814094	brown	9.659913	20.170621	0.406627	0.936944	0.88423	1.051258	6.854835	1.316097	0.415808
gene16595	CD3G	0.927439110036042	turquoise	1.880604	2.712144	2.34225	2.189097	1.924181	1.791446	2.196216	2.703468	6.588538
Sus_scrofa_newGene_1589	--	0.817602822432834	brown	1.309092	3.210881	0.240889	0.919416	0.634681	1.06431	2.066413	0.5259	1.029708
gene26319	GIMAP6	0.956900533650928	turquoise	9.510328	12.646392	12.153338	10.281825	6.788712	12.202376	9.511264	8.712712	29.071703
gene14969	RPL9	0.903084478621332	turquoise	268.631821	306.794631	166.623591	184.123364	208.197441	352.164309	138.777969	160.705444	549.09491
gene5692	RPS15A	0.988406038737175	turquoise	240.756445	139.747527	130.160864	146.749421	147.877985	197.456751	132.86232	174.593347	589.302937
Sus_scrofa_newGene_12179	--	0.826271036951391	brown	1.326535	2.81336	0.793716	1.878124	0.927289	0.818149	1.327479	0.857602	1.110191
gene10919	SNRPD2	0.973958370748294	turquoise	52.874241	30.100956	28.563021	31.63753	35.057563	44.686461	37.829195	36.525416	102.31275
gene23001	UNC5B	0.853766876066655	blue	2.488136	1.182232	0.999191	1.041573	0.566219	0.924292	1.327166	0.807529	0.659696
gene25041	GZMA	0.925713340053792	turquoise	2.873612	3.734178	5.119483	2.540615	2.505658	4.215434	3.445121	2.459687	11.09768
gene5571	ALDOA	0.961724666106088	blue	220.126347	54.242972	55.090159	56.556209	67.067862	79.657428	85.53936	71.530016	75.17731
Sus_scrofa_newGene_8389	--	0.782485453973562	turquoise	2.448815	0.824498	2.133731	0.571994	0.434615	1.050267	0.113688	0.302159	3.732037
gene21616	LOC100620198	0.938803769537105	turquoise	0.834336	0.941309	1.428435	1.165622	1.052724	1.363939	1.200546	1.140194	3.239157
gene15234	LOC100624541	0.783106345442685	blue	12.753342	1.081489	2.007102	6.111111	3.324349	8.616267	4.094918	12.203677	4.3159
gene12100	LOC110261162	0.710208419804272	green	0.450541	0.769055	0.886657	5.049153	5.015581	0.695712	5.682282	0.282153	0.884476
gene1122	FAM169B	0.836109643420958	brown	0.705822	5.96985	2.721467	3.788443	2.508505	1.644575	5.238781	1.55655	1.529013
gene16308	RAB30	0.988429531905961	blue	24.92589	0.888287	3.993476	3.806542	1.936164	6.956815	3.28453	6.585403	5.429549
gene3680	TPM4	0.978478837840333	blue	140.637935	30.483977	37.343	34.845481	35.114813	47.297685	37.374662	40.109592	39.411851
gene17766	UBE2T	0.874900721139359	turquoise	0.711355	1.107013	1.747306	1.077559	1.257561	1.197311	0.721831	1.135525	3.362807
gene3080	MDK	0.971283630525917	turquoise	12.402906	14.631887	10.637062	12.335159	6.982375	13.212062	7.537132	11.572012	34.990488
Sus_scrofa_newGene_1221	--	0.798535467059552	black	0.41943	2.391738	0.993289	2.175964	1.833051	0.958711	0.921743	0.354723	1.142276
gene12798	LY86	0.949120506933665	turquoise	6.020467	6.888773	6.034489	4.08426	5.557175	10.030272	9.718961	5.786234	24.011927
gene13864	PSTPIP1	0.958686491186721	turquoise	1.130048	0.969061	1.116292	0.651976	0.833105	1.56558	1.331121	0.645335	3.709246
gene21221	RPL22L1	0.949478070347263	turquoise	10.541323	8.428707	7.915157	9.666457	6.562356	12.116569	3.859343	5.615655	22.905798
gene23804	LOC100525613	0.914186160204617	turquoise	1.827004	0.844504	0.475337	0.485494	0.785854	1.639228	0.774047	0	3.583195
gene26430	FMC1	0.979635153412159	turquoise	7.220876	6.043205	3.288285	7.904832	5.322527	6.77702	5.646753	5.656979	21.244434
Sus_scrofa_newGene_14897	--	0.753275962674769	black	2.439571	7.112956	1.525705	20.580248	3.22328	2.383944	9.963875	4.292785	9.832532
Sus_scrofa_newGene_4166	--	0.925438447282745	brown	0.935521	3.043479	1.207856	1.452153	0.75429	1.197035	1.512418	0.999319	0.63065
